# One ligand, two regulators and three binding sites: How KDPG controls primary carbon metabolism in *Pseudomonas*

**DOI:** 10.1371/journal.pgen.1006839

**Published:** 2017-06-28

**Authors:** Rosaria Campilongo, Rowena K. Y. Fung, Richard H. Little, Lucia Grenga, Eleftheria Trampari, Simona Pepe, Govind Chandra, Clare E. M. Stevenson, Davide Roncarati, Jacob G. Malone

**Affiliations:** 1 John Innes Centre, Norwich Research Park, Colney Lane, Norwich, United Kingdom; 2 Istituto Pasteur- Fondazione Cenci Bolognetti, Dipartimento di Biologia e Biotecnologie ‘‘C. Darwin”, Sapienza Universita`di Roma, Roma, Italy; 3 University of East Anglia, Norwich Research Park, Norwich, United Kingdom; 4 Alma Mater Studiorum - University of Bologna, Department of Pharmacy and Biotechnology – FaBiT, Bologna, Italy; Universidad de Sevilla, SPAIN

## Abstract

Effective regulation of primary carbon metabolism is critically important for bacteria to successfully adapt to different environments. We have identified an uncharacterised transcriptional regulator; RccR, that controls this process in response to carbon source availability. Disruption of *rccR* in the plant-associated microbe *Pseudomonas fluorescens* inhibits growth in defined media, and compromises its ability to colonise the wheat rhizosphere. Structurally, RccR is almost identical to the Entner-Doudoroff (ED) pathway regulator HexR, and both proteins are controlled by the same ED-intermediate; 2-keto-3-deoxy-6-phosphogluconate (KDPG). Despite these similarities, HexR and RccR control entirely different aspects of primary metabolism, with RccR regulating pyruvate metabolism (*aceEF*), the glyoxylate shunt (*aceA*, *glcB*, *pntAA*) and gluconeogenesis (*pckA*, *gap*). RccR displays complex and unusual regulatory behaviour; switching repression between the pyruvate metabolism and glyoxylate shunt/gluconeogenesis loci depending on the available carbon source. This regulatory complexity is enabled by two distinct pseudo-palindromic binding sites, differing only in the length of their linker regions, with KDPG binding increasing affinity for the 28 bp *aceA* binding site but decreasing affinity for the 15 bp *aceE* site. Thus, RccR is able to simultaneously suppress and activate gene expression in response to carbon source availability. Together, the RccR and HexR regulators enable the rapid coordination of multiple aspects of primary carbon metabolism, in response to levels of a single key intermediate.

## Author summary

Here we show how *Pseudomonas* controls multiple different primary carbon metabolism pathways by sensing levels of KDPG, an Entner Doudoroff (ED) pathway intermediate. KDPG binds to two highly similar transcription factors; the ED regulator HexR and the previously uncharacterised protein RccR. RccR inversely controls the glyoxylate shunt, gluconeogenesis and pyruvate metabolism, suppressing the first two pathways as pyruvate metabolism genes are expressed, and vice versa. This complex regulation is enabled by two distinct RccR-binding consensus sequences in the RccR regulon promoters. KDPG binding simultaneously increases RccR affinity for the glyoxylate shunt and gluconeogenesis promoters, and releases repression of pyruvate metabolism. This elegant two-regulator circuit allows *Pseudomonas* to rapidly respond to carbon source availability by sensing a single key intermediate, KDPG.

## Introduction

Soil-dwelling *Pseudomonas* spp. are exposed to a complex and dynamic physical and chemical environment, and must constantly adapt their cell physiology by changing the expression patterns of membrane proteins, secreted small molecules, and enzymes [1]. This ability to gauge the surroundings and modulate gene expression accordingly is crucial for effective environmental adaptation [2]. Therefore, the capacity of *Pseudomonas* to prosper in different niches depends not only on the acquisition of nutrients and effective resistance to external stresses, but also on the effective deployment of sensory proteins, signal-transduction pathways and transcriptional regulators [3]. While much research into niche colonisation by microbes has focussed on phenotypic adaptations such as motility, biofilm formation and stress resistance [4, 5], the remodelling of central metabolism to optimally respond to the external environment is a critical and understudied trait.

The efficient coordination of carbon uptake and flux through the primary metabolic pathways with the nutrient availability of the surroundings is key to the successful colonisation of most ecological niches [6], and relies on multilevel control of the expression and activity of proteins involved in primary metabolism [7]. A range of transcriptional factors orchestrates bacterial gene expression in response to carbon source availability, generating a sophisticated control network for both catabolic and anabolic metabolism [8]. Transcription factors belonging to the RpiR family control the expression of enzymes involved in carbon metabolism [9]. Members of this family are characterized by a helix-turn-helix DNA-binding domain at the N-terminus and a sugar isomerase domain (SIS) at the C-terminus [10, 11]. In *Pseudomona*s, the RpiR regulator HexR controls the uptake and the catabolism of glucose, modulating the expression of glucose phosphorylative pathway and Entner-Doudoroff (ED) pathway genes [12–14]. HexR gene targets are grouped in two well-conserved operons containing genes for glucose catabolism and transport [14]. HexR binds as a dimer to the pseudo-palindromic DNA consensus sequence 5’- TTGTN7-8ACAA-3’, found in the *zwf*/*hexR* and *edd*/*gap-1* intergenic regions, and represses gene transcription in its apo-form. When glucose is present, it is imported and metabolised via several steps to 2-keto-3-deoxy-6-phosphogluconate (KDPG), an exclusive metabolic intermediate of the ED pathway. HexR binds KDPG via its SIS domain, signalling glucose availability and causing HexR to dissociate from DNA, inducing expression of its gene targets and stimulating glucose uptake and metabolism [14].

Downstream of glycolysis, the Krebs cycle is responsible for the complete oxidation of acetyl-CoA to CO_2_ and provides intermediates that are necessary for the production of amino acids and other cellular macromolecules. The pools of these metabolic intermediates must be constantly resupplied to maintain them at sufficient levels for metabolism and growth. For this reason it is important that flux through and around the Krebs cycle is exquisitely controlled to make best use of available carbon sources [15]. During Krebs cycle operation, acetyl-CoA condenses with oxaloacetate to form citrate, which is, following a complete turn of the cycle, reconverted to oxaloacetate. Each turn of this cycle involves the loss of two molecules of CO_2_. When acetyl-CoA is the only available carbon source, classical Krebs cycle operation cannot assimilate carbon. Consequently, when acetate or fatty acids are the primary source of carbon and energy, many bacterial species including *Pseudomonas* [16, 17] activate a specific anaplerotic pathway, called the Glyoxylate shunt [18]. The Glyoxylate shunt is common among microorganisms and higher plants [19, 20], and diverts part of the carbon flux at isocitrate [18, 21]. The key enzymes of this pathway are isocitrate lyase (ICL), which converts isocitrate to glyoxylate, and malate dehydrogenase, which condenses a molecule of glyoxylate with an acetyl-CoA to produce malate and succinate. This ensures the replenishment of the metabolic intermediates required for the biosynthesis of cellular components. The only regulatory mechanism for the Glyoxylate shunt known to date involves the reversible phosphorylation of isocitrate dehydrogenase (IDH) [22–24]. When bacteria grow on two-carbon compounds, such as acetate, IDH phosphorylation inactivates this enzyme and forces carbon flux through the shunt pathway.

*P*. *fluorescens* is a common soil bacteria that non-specifically colonizes plant roots, and can improve plant health through nutrient recycling, pathogen antagonism, and inducing plant defence responses [3]. The rhizosphere environment is both highly complex and attractive to microbial life. Plant roots continuously produce and secrete compounds in the rhizosphere environment (including ions, amino acids, organic acids, sugars, phenolics and other secondary metabolites) [25, 26], and many microorganisms are attracted to these exuded nutrients [27]. Successful root colonisation depends on both the coordinated expression of factors involved in phenotypes such as motility and biofilm formation [5, 28] and on the adaptive remodelling of central metabolism [4, 29]. Recently, an uncharacterised member of the RpiR family; *rccR* (*PFLU6073*), was identified in an *In Vivo Expression Technology* (IVET) screening experiment for loci involved in *P*. *fluorescens* plant interactions [30]. RccR shares a very high amino-acid sequence identity (43% identical) with HexR, and both transcription factors play important roles in wheat rhizosphere colonisation and growth on defined carbon sources. Furthermore, the HexR/RccR regulon is widespread among the pseudomonads and in several other bacterial genera. This prompted us to investigate the contributions of these ‘twin’ transcription factors to environmental adaption in more detail.

We first examined the role of HexR in *P*. *fluorescens* during the wheat rhizosphere colonisation, confirming its function as a regulator of glucose metabolism, consistent with earlier data [14]. Next, we characterized RccR, and identified it as a master regulator of pyruvate metabolism, the glyoxylate shunt and gluconeogenesis. RccR binds to two distinct, pseudo-palindromic binding sites in the promoter regions of its target genes, which share a binding sequence but differ in the length of the linker region. RccR shares a ligand; the ED pathway intermediate 2-dehydro-3-deoxy-phosphogluconate (KDPG), with HexR, reinforcing the regulatory connection between the two proteins. Furthermore, ligand binding increases RccR affinity for one binding site but decreases it for the other, enabling a sophisticated transcriptional response to a single metabolic intermediate. We propose that the coordinated activity of HexR and RccR tightly controls the remodelling of central metabolism in response to intracellular KDPG levels, maximizing bacterial proliferation and fitness, and optimising enzyme synthesis to most effectively respond to nutrient availability in the environment.

## Results

### RccR and HexR are highly similar proteins and are both important for plant colonisation

**Fig 1:**
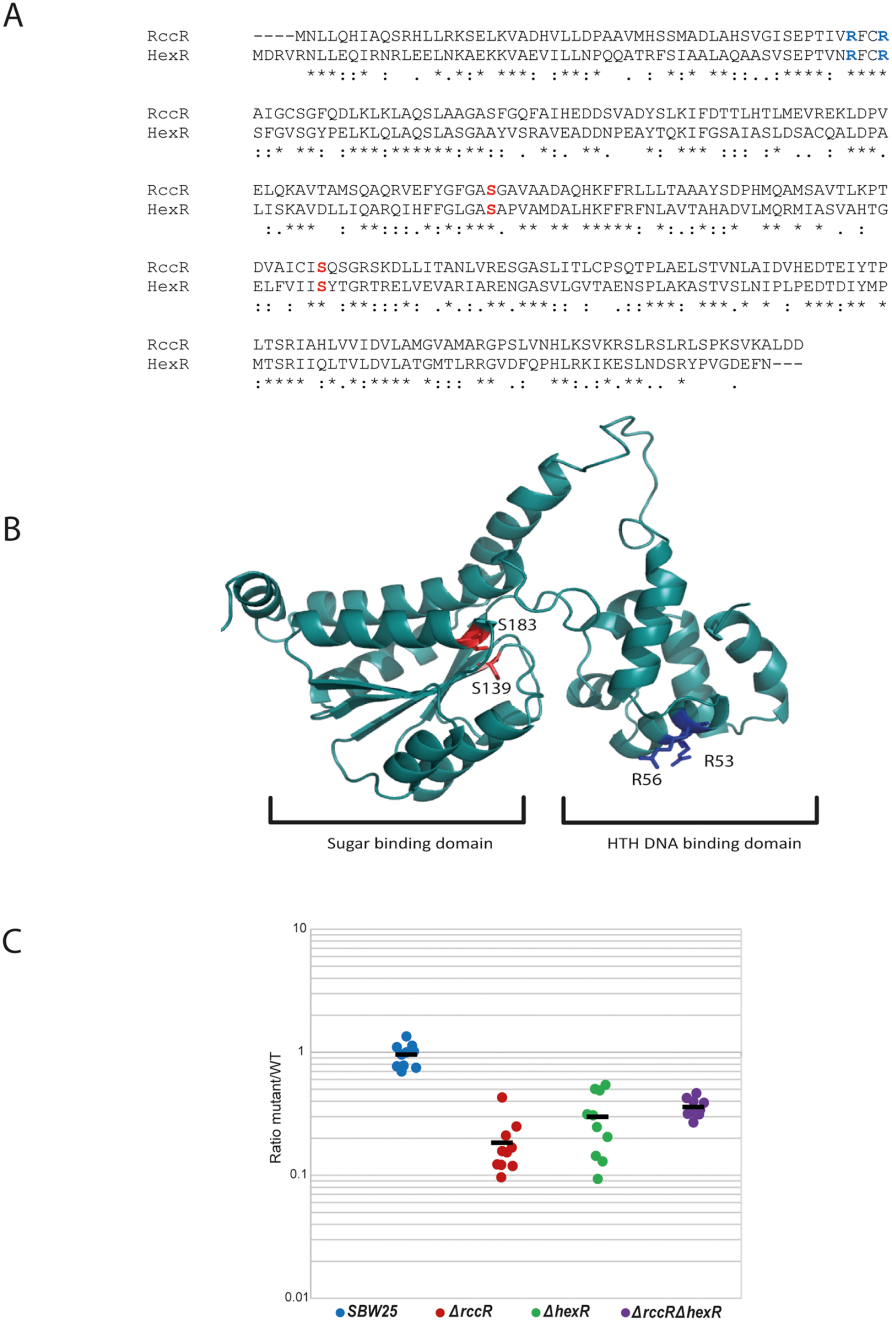
RccR and HexR are highly similar and important for wheat rhizosphere colonisation. **1A**: Sequence alignment for *rccR* and *hexR* from *P*. *fluorescens* SBW25. Important amino acid residues for DNA and ligand interactions are marked in blue and red respectively. **1B**: 3D homology model of the RccR protein structure. Arg-53 and -56 (blue) are the predicted DNA interaction partners in the helix-turn-helix domain. Ser-139 and -183 (red) are located in the predicted effector binding site. **1C**: Rhizosphere colonisation competition assays. The graph shows the ratio of SBW25 WT or *ΔrccR*/*ΔhexR* mutants to WT-*lacZ* colony forming units (CFU) recovered from the rhizospheres of wheat plants seven days post-inoculation. Each dot represents the ratio of CFUs recovered from an individual plant. In each case, differences between SBW25 and *ΔrccR* or *ΔhexR* strains are statistically significant (p < 0.05, Mann-Whitney U test).

The *P*. *fluorescens* SBW25 *rccR* (*PFLU6073*) gene encodes an uncharacterised member of the RpiR family of transcriptional regulators. Following the observation that *rccR* seems to play a role in the plant environment [30] we decided to examine the locus in more detail. SBW25 RccR has a striking degree of similarity (43% amino acid identity, over 70% similarity) to the central metabolic regulator HexR. Examination of the primary structure of both proteins indicated that the key residues of the DNA binding motif (R57, R60), and ligand binding site (S139, S183) are conserved in both cases (Fig 1A). To identify any differences between the two proteins, molecular models of both were produced based on four published crystal structures of transcriptional regulators (PDB files: 3sho, 2o3f, 4ivn and 3iwf). Both models were essentially identical, with no obvious differences in binding sites or residue conformation emerging from this analysis (Fig 1B).

Despite their highly similar predicted structures, *hexR* does not appear in the initial sugar beet IVET dataset [30], suggesting distinct regulatory roles for RccR and HexR. To test this, we examined the significance of both *rccR* and *hexR* to rhizosphere colonisation and plant interaction. Single and double *rccR*/*hexR* mutants were produced in *P*. *fluorescens* SBW25, and a series of competitive colonisation assays performed with *lacZ*-labelled WT SBW25. After seven days, significantly fewer *ΔrccR*, *ΔhexR* and *ΔrccRΔhexR* colony forming units (CFUs) were recovered from model rhizospheres compared with the WT-*lacZ* competitor (Fig 1C), indicating that both genes are similarly important for competitive growth in the plant root environment.

### HexR and RccR are important for bacterial growth in different carbon sources

**Fig 2:**
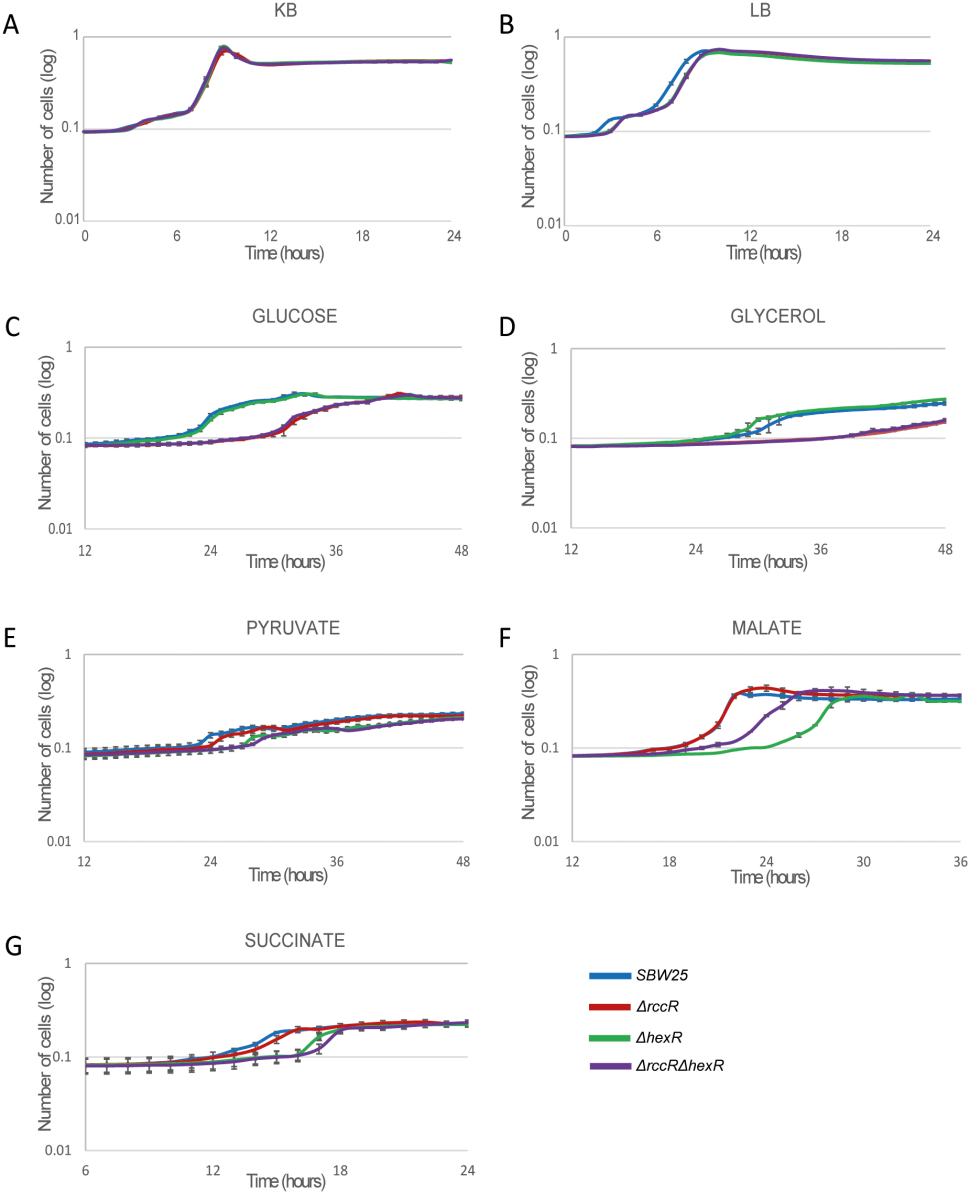
Growth curves for SBW25 WT and *ΔrccR*, *ΔhexR*, and *ΔrccRΔhexR* mutants. **2A**: Growth was measured in KB and **2B**: LB rich media as well as in **2C**: M9 0.4% glucose, **2D**: M9 0.4% glycerol, **2E**: M9 0.4% pyruvate, **2F**: M9 0.4% acetate and **2G**: M9 0.4% succinate. Marked differences in growth rate were seen between WT and *ΔrccR* in glucose (**C**) and glycerol (**D**), and between WT and *ΔhexR* mutants in pyruvate (**E**), acetate (**F**), and succinate (**G**). Experiments were repeated at least three times independently and a representative plot is shown in each case.

We examined the responses of the *rccR and hexR* mutants to different nutrient conditions by testing their ability to grow in rich, complex and defined minimal media containing different sources of carbon. We observed little effect of *rccR/hexR* gene deletion in rich media; no growth-rate difference in KB, and a slight delay in log phase entry for both mutants in LB (Fig 2A/2B). Conversely, deletion of *rccR* resulted in severely compromised bacterial growth in minimal medium with either glucose or glycerol as the sole carbon source (Fig 2C/2D). Deletion of *hexR* on the other hand affected growth in media containing pyruvate, acetate or succinate, (Fig 2E, 2F and 2G). These growth defects were dominant and not additive: the *ΔrccR/ΔhexR* double mutant displayed an *rccR*-mutant phenotype in glycerol and glucose, and a *hexR-*mutant phenotype in pyruvate, acetate, or succinate (Fig 2). Interestingly, an exception is the bacteria growth in acetate, in which the double mutant shows an intermediate ability to grow compared to the wild type and the single mutants (Fig 2F). Despite their marked sequence and structural similarities, the activities of RccR/HexR are clearly distinct, with their relative importance varying markedly depending on the available carbon sources.

### *P*. *fluorescens* HexR is a regulator of glucose metabolism

**Fig 3:**
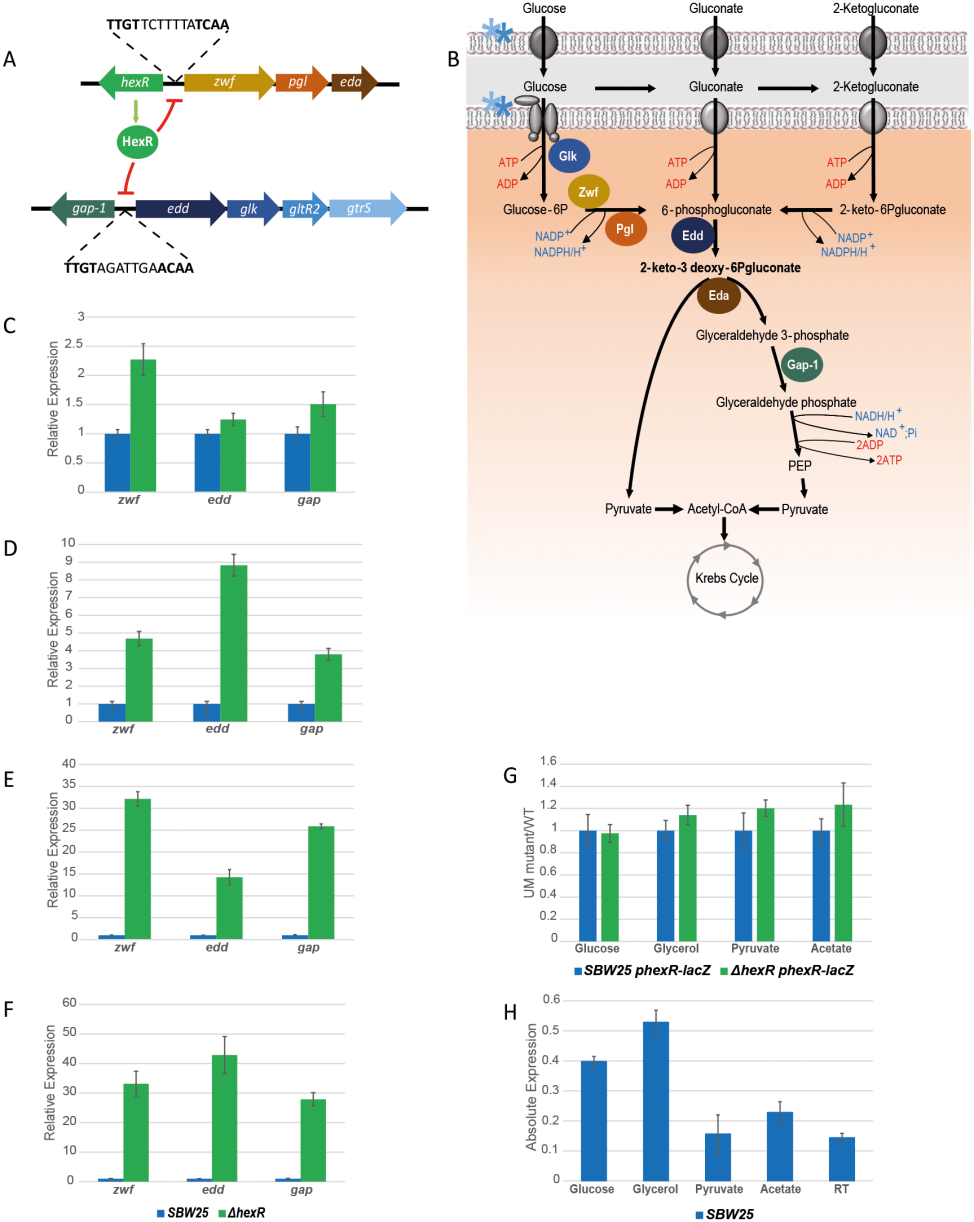
HexR controls the Entner-Doudoroff pathway in *P*. *fluorescens*. **3A**: Schematic organisation of the HexR gene targets. HexR binds to a DNA consensus sequence in the intergenic regions between the *zwf/pgl/eda* operon and *hexR* genes, and between the *edd/glk/gltR2/gltS* operon and the *gap-1* gene. HexR negatively regulates expression of these gene targets, but not of itself. **3B**: The HexR regulon. HexR gene targets are involved in the glucose phosphorylative and Entner-Doudoroff pathways in *P*. *fluorescens*. Glk: glucokinase; Zwf: glucose 6-P dehydrogenase; Pgl: 6-phosphogluconolactonase; Edd: 6-phosphogluconate dehydratase; Gap-1: glyceraldehyde 3-phosphate dehydrogenase; the blue and light blue stars indicate activation of the glucose transport system, which is positively regulated by the transcriptional regulators GltR2 and GltS. **3C**: *zwf*, *edd*, and *gap* gene expression in glucose, **3D**: in glycerol, **3E**: in pyruvate and **3F**: in acetate in the *hexR* mutant background relative to WT (qRT-PCR data). **3G**: *hexR* promoter activity in SBW25 Δ*hexR* relative to WT, determined by β-gal assays tested in glucose, glycerol, pyruvate and acetate conditions. **3H**: SBW25 *hexR* gene expression determined by qRT-PCR after media exchange and 30 min growth in glucose, pyruvate or Root Solution (RS; media without carbon sources, used as a negative control).

Previous studies have shown that HexR is a transcriptional repressor of the glucose phosphorylative and Entner-Doudoroff pathways in *P*. *putida* and *P*. *aeruginosa* [14, 31]. HexR represses the expression of its gene targets by binding to the specific consensus sequence 5’- TTGT-N_7-8_-ACAA-3’ (Fig 3A). To verify the HexR regulatory system (Fig 3B) in *P*. *fluorescens*, we first examined the intergenic regions between the p-*edd* and p-*gap-1*, and between the p-*zwf* and p-*hexR* promoters in the SBW25 genome for the HexR consensus binding sequence. As expected, both intergenic regions contained HexR binding sites. Next, qRT-PCR experiments were performed to examine the expression of the *gap-1*, *edd* and *zwf* genes in WT and *ΔhexR* backgrounds, for bacteria grown in minimal media containing different, single carbon sources. While little difference in expression of these genes was observed between WT and *ΔhexR* for bacteria grown in glucose minimal media (Fig 3C), the expression profile of the Δ*hexR* mutant significantly diverged from WT when the strains were grown in glycerol, pyruvate or acetate media. Under these conditions, expression of *gap-1*, *edd* and *zwf* significantly increased in the Δ*hexR* background (Fig 3D, 3E and 3F). These results suggest the strong repression of *gap-1*, *edd* and *zwf* genes by *P*. *fluorescens* HexR, particularly under conditions where KDPG is unable to accumulate, and consistent with earlier data [14].

To test whether HexR represses its own expression, we constructed a p-*hexR*-*lacZ* transcriptional reporter plasmid (pME-*hexR*) and performed β-galactosidase assays to study the promoter activity of *hexR* in WT SBW25 and the *ΔhexR* mutant, grown in either minimal glucose or pyruvate medium. We observed no autoregulation of *hexR* promoter activity when bacteria were grown in any of the tested media (Fig 3G). However, qRT-PCR data showed a higher *hexR* expression level when grown with glucose or glycerol, but not with pyruvate or acetate as the sole carbon source (Fig 3H).

### RccR is a regulator of the glyoxylate shunt and gluconeogenesis pathways

**Fig 4:**
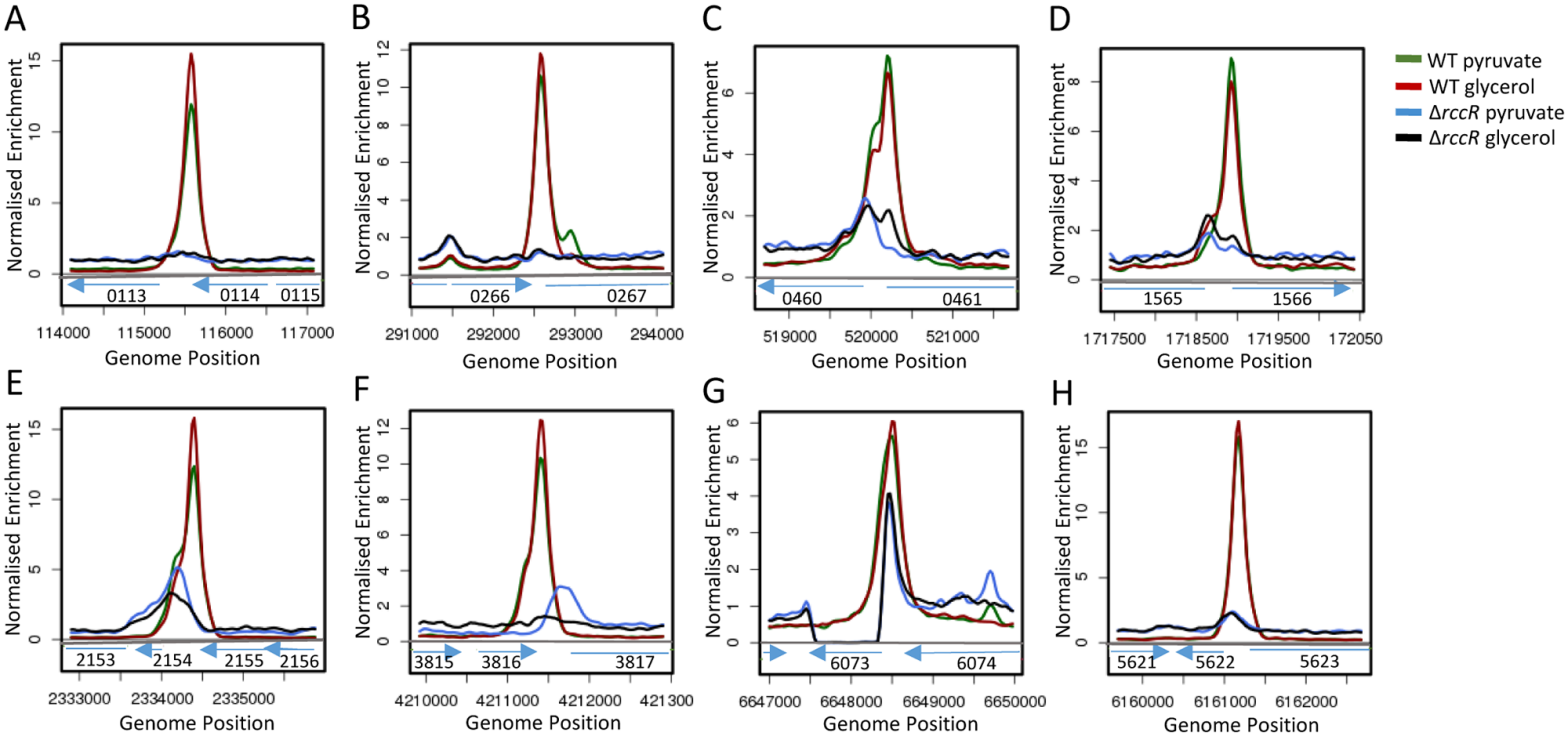
Mapped reads from the RccR ChIP-seq experiment. **4A-H**: Locations of genes and operons of interest are shown below each peak. Blue arrows indicate the direction of gene transcription and *PFLU* gene numbers are indicated in each case. Relative scales are indicated for each panel as well as the gene position in the SBW25 genome. Green and red peaks denote the SBW25 WT datasets, while blue and black show data for the *ΔrccR* mutant strain. Green and black lines indicate bacterial growth in glycerol, while red and blue indicate bacterial growth in pyruvate.

Despite the high predicted structural similarity between HexR and RccR, their markedly different impacts on SBW25 growth suggest that these proteins control independent transcriptional regulons. To characterise the RccR regulon, we performed a ChIP-seq assay using a polyclonal RccR antiserum on SBW25 WT/*ΔrccR* strains grown in minimal pyruvate and glycerol media. While little difference was seen between the two media conditions, based on a stringent peak-calling analysis we were able to identify 8 RccR binding sites from this experiment (Fig 4), including one in the *rccR* promoter region itself (Fig 4G). The peaks identified in our RccR ChIP-seq assays corresponded to strongly enriched regions relative to the respective Δ*rccR* controls. All 8 peaks were localized in intergenic regions, upstream of one or more genes (Table 1). To verify that the identified binding sites are associated with RccR-mediated regulation, we extracted RNA from SBW25 WT and *ΔrccR* grown in M9 glycerol, and performed qRT-PCR assays for each member of the putative RccR regulon (except *rccR*). Where a putative binding site lay between two divergent genes, both targets were tested and RccR regulation shown unambiguously for one of them (S1 Fig). In each case, we saw a marked increase of expression in *ΔrccR* relative to WT for only one of the two possible targets (S1 Fig), indicating RccR repression. The RccR regulon includes enzymes involved in gluconeogenesis (phosphoenolpyruvate carboxykinase (*pckA*) and glyceraldehyde-3-phosphate dehydrogenase (*gap*), NADPH/NAD^+^ redox balance (NAD(P) transhydrogenase (*pntAA*/*PFLU0112*/*pntB*), acetyl-CoA production (*aceE/F*) and the glyoxylate shunt pathway (isocitrate lyase (*aceA*) and malate synthase G (*glcB*)) (Table 1).

**Table 1:** RccR gene targets.

RccR gene targets	Description
PFLU0113 *pntAA*	NAD(P) transhydrogenase subunit α1
PFLU0267 *pckA*	phosphoenolpyruvate carboxykinase
PFLU0460 *aceE*	pyruvate dehydrogenase subunit E1
PFLU1566 *gap*	glyceraldehyde-3-phosphate dehydrogenase
PFLU2154	hypothetical protein
PFLU3817 *aceA*	isocitrate lyase
PFLU5623 *glcB*	malate synthase G
PFLU6073 *rccR*	transcriptional factor

**Fig 5:**
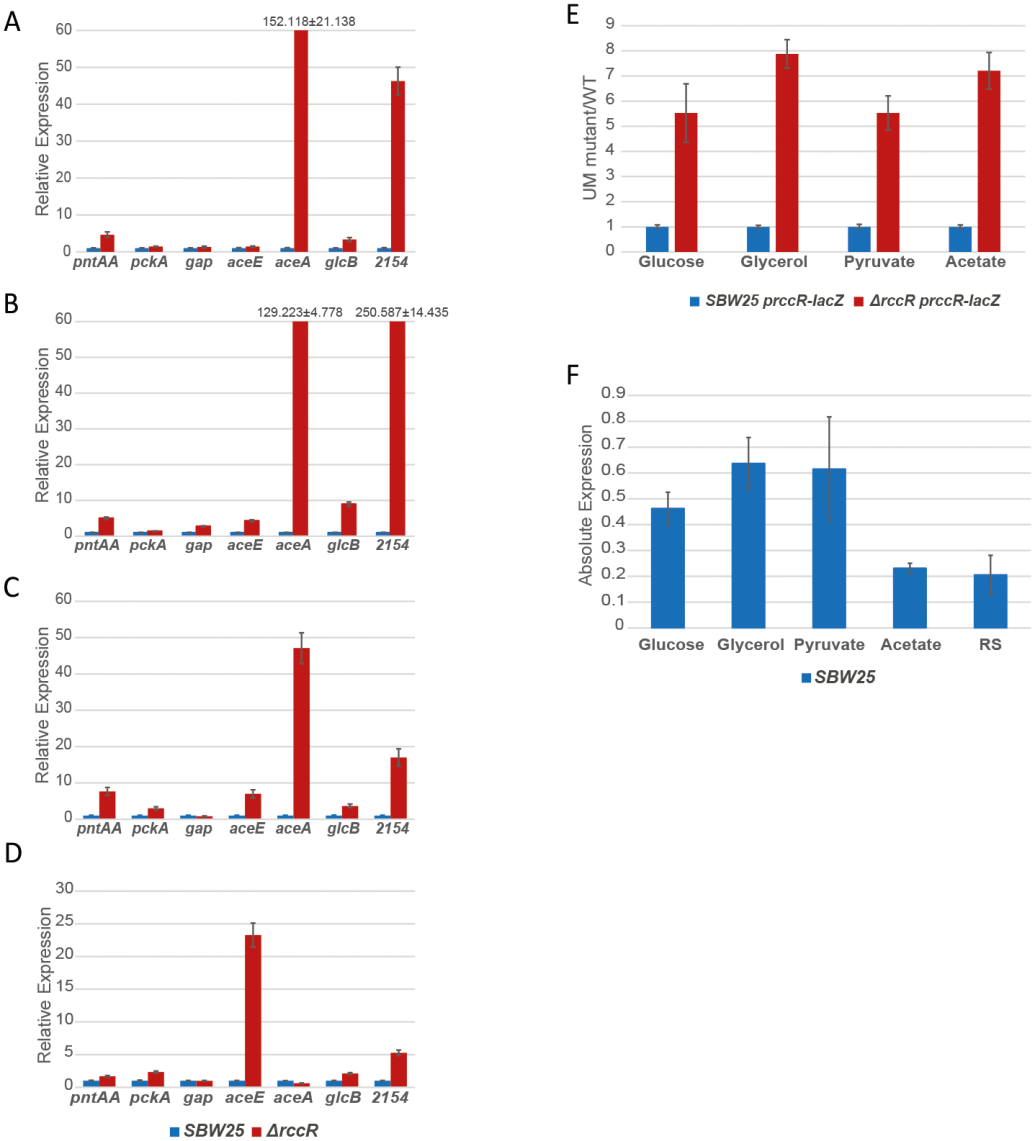
RccR controls expression of pyruvate metabolism, gluconeogenesis and the glyoxylate shunt. **5A-C**: RccR gene target expression determined by qRT-PCR. Data are shown for SBW25 *ΔrccR* relative to WT in **5A**: glucose media, **5B**: glycerol media, **5C**: pyruvate media, and **5D**: acetate media. **5E**: *rccR* promoter activity determined by β-gal assay in glucose, glycerol, pyruvate and acetate media conditions. Data are shown for the SBW25 *ΔrccR* background relative to WT. **5F**: SBW25 *rccR* gene expression determined by qRT-PCR after media exchange and 30 min growth in glucose, glycerol, pyruvate, acetate or Root Solution (RS; media without carbon sources, used as a negative control).

Most members of the RccR regulon have well-understood enzymatic functions in carbon metabolism. The notable exception, *PFLU2154*, encodes a hypothetical protein of unknown function. This gene shows a similar expression profile to *aceA*, with particularly strong RccR repression seen in glycerol, and much less effect in acetate (Fig 5A/5D). This suggests that this gene may play an important role in two-carbon substrate catabolism. To test this, a non-polar *PFLU2154* deletion mutant was produced and its ability to grow on different carbon sources was tested. Consistent with both our hypothesis and previous data, the only condition in which the mutant showed a growth defect was in acetate medium (S2A/S2H Fig). We further examined the ability of the Δ*PFLU2154* mutant to competitively colonize wheat roots. No significant differences in colonisation were seen for the mutant compared to WT SBW25 (S2I Fig).

To more closely examine the role of RccR in SBW25 gene regulation, we extracted additional RNA samples from WT and *ΔrccR* cultures grown in minimal media containing glucose, pyruvate or acetate, and used qRT-PCR to probe expression of the target genes in Table 1. The impact of *rccR* deletion on gene expression broadly matched its impact on growth in different carbon sources. When grown in glucose or glycerol as the sole carbon source, RccR tightly repressed the *aceA* and *glcB* genes, indicating inhibition of the glyoxylate shunt pathway. On the other hand, much less control was seen for *aceE/F* gene expression (Fig 5A/5B). When bacteria grew in minimal pyruvate the *aceA* and *glcB* genes are still repressed but to a lesser (albeit still significant) extent than in glucose and glycerol. Again, little repression was seen for *aceE/F* (Fig 5C). A much more striking shift was seen for strains grown in minimal acetate media (Fig 5D). In this case, no inhibition was observed for either *aceA* or *glcB* gene expression, suggesting activation of the glyoxylate bypass in these conditions. While *rccR* deletion in acetate media had little effect on most tested genes, the *aceE/F* locus was strongly expressed (Fig 5D), indicating a strong down-regulation of pyruvate dehydrogenase production under conditions where pyruvate metabolism is no longer required and acetyl-CoA may be produced directly from acetate.

To probe RccR control of its own transcription, we cloned the *rccR* promoter region into the pME*lacZ* plasmid, and examined the impact of *rccR* deletion on β-gal activity in different carbon conditions. As expected, RccR repressed its own expression, with increased β-gal activity in the Δ*rccR* strain in every condition tested: glucose, glycerol, pyruvate and acetate minimal media (Fig 5E). Consistent with this, qRT-PCR data showed an approximately constant *rccR* gene expression when grown in glucose, glycerol or pyruvate medium, although a lower expression was observed with acetate as the sole carbon source (relative to carbon-free rooting solution) (Fig 5F).

### RccR recognises two distinct pseudo-palindromic binding sequences

**Fig 6:**
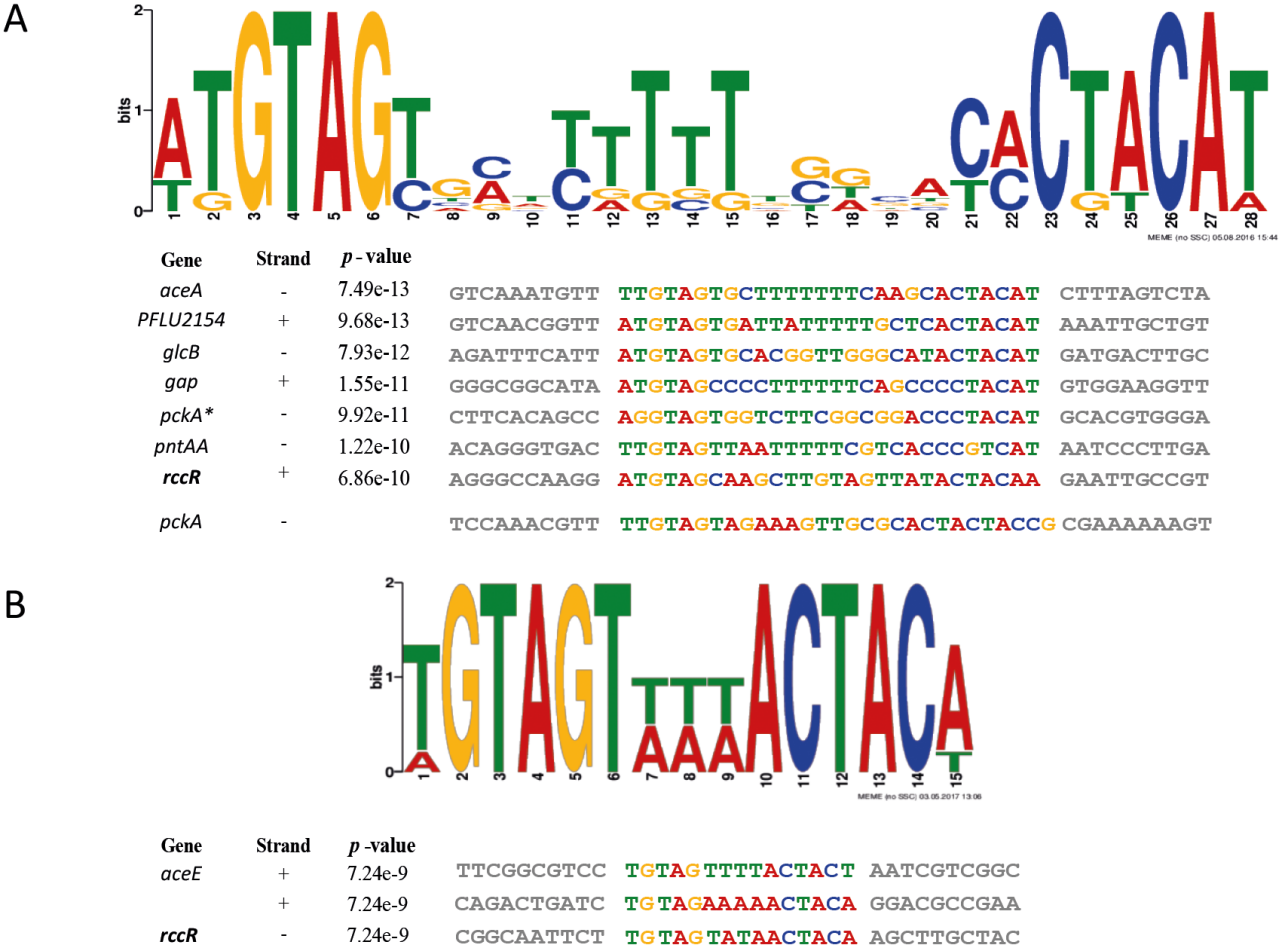
RccR has two related, pseudo-palindromic binding sequences. **6A**: The predicted 28 bp RccR DNA-binding site identified by MEME analysis. This consensus is generated from the sequences identified in each RccR binding region, including the binding site located 292 bp after the *pckA* start codon (indicated with an *). The relative p- values of each RccR binding sites is indicated alongside the name of the RccR gene target in each case. The manually-identified 29 bp site upstream of *pckA* is also shown. **6B**: The predicted 15 bp RccR DNA-binding site identified by MEME analysis. The sequences found in the upstream regions of *aceE* and *rccR* are indicated with the relative p-values of each. The *aceE* upstream region contains two slightly different RccR binding sites 68 bp apart (TGTAGTTTTACTACT and TGTAGTAAAACTACA), both of which were used to generate the consensus sequence.

The sequence surrounding the eight RccR ChIP-seq binding sites was analysed for potential binding motifs by MEME. A 28 bp pseudo-palindromic consensus sequence was found in 7 sites, with an E-value of <1.8e^-10^ and *p-*value <0.05 for each sequence (Fig 6A). The 28 bp pseudo-palindrome was not identified in the upstream regions of *pckA* or *aceE*. The ChIP-seq dataset for pyruvate grown cells contains an additional, smaller peak inside the *pckA* open reading frame (Fig 4B), corresponding in location to a second 28 bp pseudo-palindrome located around 300 bp after the start codon (Fig 6A) and suggesting an additional layer of RccR-regulation for this gene. Subsequent manual analysis of the *pckA* promoter located a 29 bp RccR-pseudo-palindrome with an additional base-pair in the linker region (Fig 6A), likely accounting for the failure of MEME to identify it.

In the case of *aceE*, the upstream region contains two copies of a single 15 bp pseudopalindromic sequence, separated by 68 bp. These two sequences (15 bp and 28 bp) contain the same palindromic repeat (TGTAGT/ACTACA), with the only difference between them the length of the inter-repeat region (Fig 6B). The ChIP-seq data for the *aceE* upstream region contains a distinctive double peak in both tested conditions (Fig 4C), suggesting that RccR binding occurs to both 15 bp sites in the *aceE* upstream region. Interestingly, in the *rccR* upstream region we identified a degenerate 28 bp consensus sequence, wholly containing the shorter 15 bp sequence (Fig 6B).

**Fig 7:**
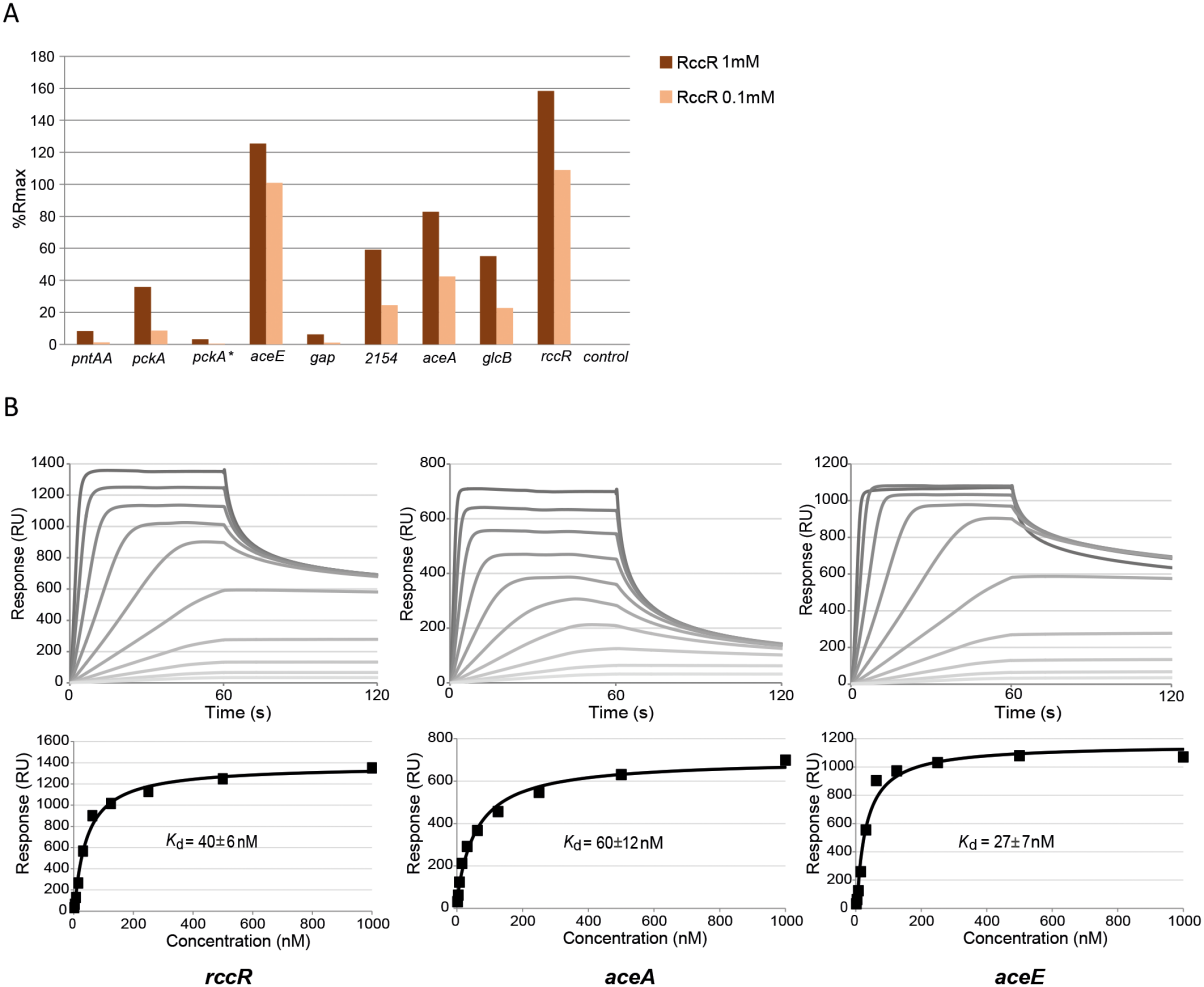
RccR binds the DNA consensus binding site of its targets. **7A**: SPR experiments measuring the biomolecular interactions between the RccR protein and indicated DNA consensus sequences. Percentage of normalized response (%Rmax) of RccR (1μM and 0.1 μM concentrations) binding the consensus sequences found by MEME and manual sequence analysis alongside a random sequence DNA control. %Rmax indicates the experimental RccR binding values (Response registered from the SPR machine) normalized on the maximal response (R_max_) that can be potentially reached when all ligand binding sites (DNA) are occupied by the analyte (RccR protein). **7B**: Sensorgrams (up) and fitting (down) curves showing RccR affinity to *aceE*, *aceA* and *rccR* consensus sequences.

To verify the RccR binding on these predicted consensus sequences, we performed ReDCaT SPR assays [32] using C-terminal His-tagged purified protein (RccR-His). We confirmed the interaction between RccR-His and the predicted RccR binding sites for every tested target, with weaker RccR binding seen for the *pckA*, *pntAA* and *gap* sequences compared to the other RccR targets (Fig 7A). We saw only very weak RccR binding to pckA*, which combined with its intragenic location suggests that this sequence may not represent a relevant RccR binding target. Next, in order to more closely analyse RccR binding to the two distinct consensus sequences, we performed further SPR experiments to study RccR-His binding affinity to *aceE*, *aceA*, and *rccR*. These analyses showed that RccR binds to the *rccR* binding site, and to a single copy of the 15 bp *aceE* sequence (*aceE* > *rccR*), with considerably higher affinity than to the *aceA* sequence (Fig 7B).

**Fig 8:**
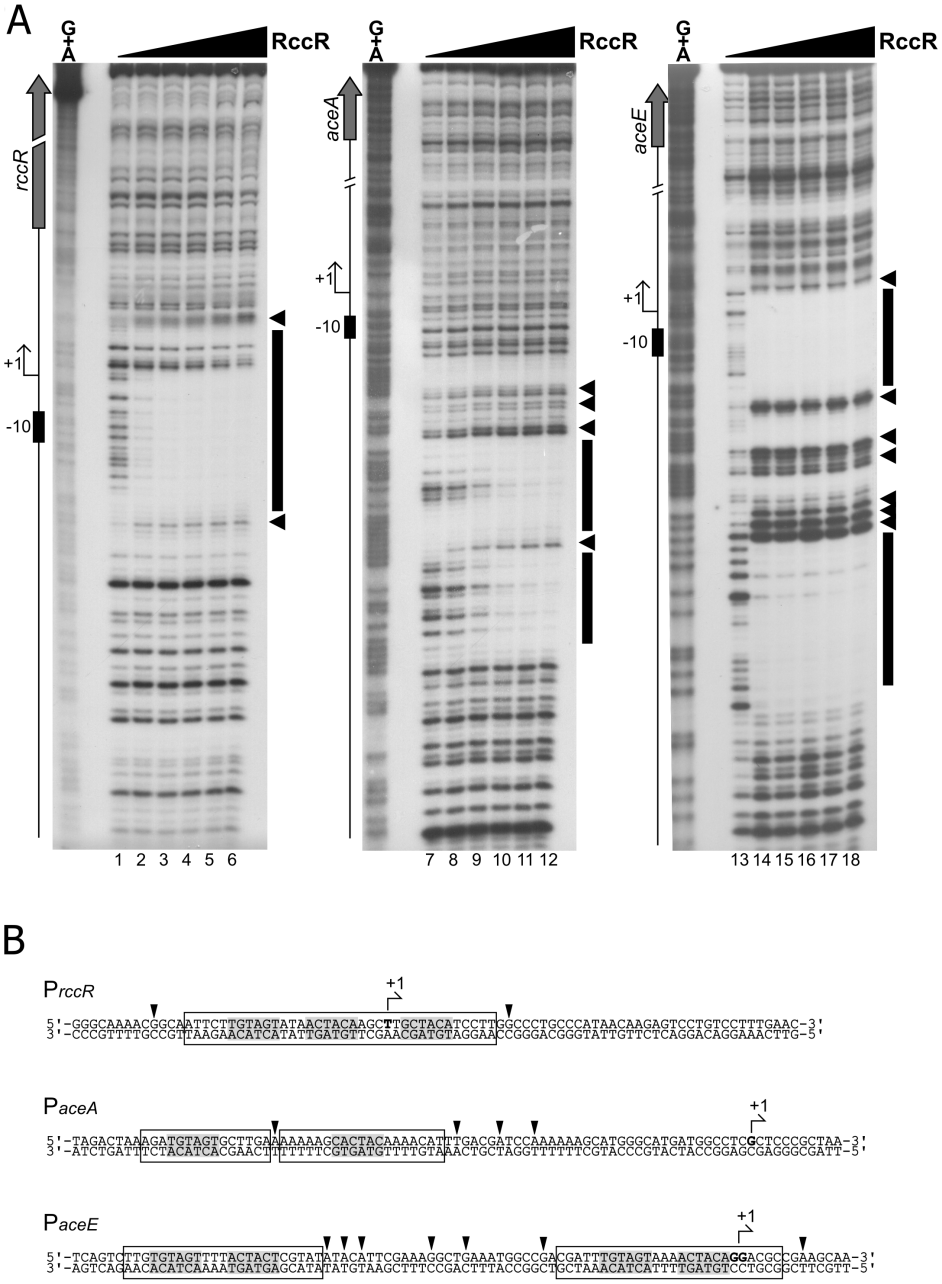
RccR binds the 28bp and the 15bp binding sites. **8A**: DNaseI footprinting panel of RccR on *rccR*, *aceA*, *aceE* promoters. Radiolabelled promoter probes were incubated with increasing concentrations of purified RccR-His (0, 10, 20, 40, 80, 160 nM of RccR-His from left to right in each panel) before DNaseI digestion and DNA purification. Recovered DNA fragments were subjected to electrophoretic separation along with a Maxam and Gilbert G+A sequence reaction ladder (leftmost lane of each autoradiograph). On the left of each autoradiograph, a schematic representation of the genomic region is reported, with symbols as follows: block arrow represents the coding sequence, bent arrow represents the transcriptional start site identified in this study (S3 Fig), while black box indicates the -10 promoter element. Protected regions are highlighted by a black box on the right of each autoradiograph, while DNaseI hypersensitive sites are evidenced by black arrowheads. **8B**: mapping of the RccR binding sites on the *rccR*, *aceA* and *aceE* promoter regions. Arrowheads denote hypersensitive sites, protected regions are included in open boxes, and conserved pseudopalindromic sequences are highlighted in light grey. Bent arrow indicates the transcriptional start site identified in this study (S3 Fig) and the first transcribed nucleotide is in bold.

In order to verify and map the predicted RccR binding sites, we first performed 5’ RACE analysis of the strongly binding DNA targets to identify transcriptional start sites (S3 Fig). Next, we performed DNaseI footprinting experiments on the *rccR*, *aceA* and *aceE* promoter regions using the RccR-His protein at an nM concentration range. Fig 8A indicates that RccR specifically binds to all the tested targets. A single binding region was observed for *rccR* and *aceA* (Fig 8A, lanes 1 to 6 and lanes 7 to 12, respectively), with the latter binding site containing a central DNaseI hypersensitive band. Notably, for the *aceE* fragment, we found two distinct binding sites flanking a DNA region rich in hypersensitive bands, suggestive of DNA bending (Fig 8A, lanes 13 to 18). Under our experimental conditions, RccR seems to recognize both *aceE* and *rccR* with very high relative binding affinity (*aceE* > *rccR*, with full protection observed at 10 nM and 20 nM RccR-His on *aceE* and on *rccR*, respectively), while *aceA* is bound with a lower affinity (full protection at 80 nM RccR-His). Given the apparent binding cooperativity between the two *aceE* 15 bp sites, it is possible that the binding affinity of RccR for the *aceE* upstream region *in vivo* is even higher than that calculated by SPR for a single *aceE* site (Fig 7B). The RccR binding sites for all three sequences were precisely mapped (reported in Fig 8B) and shown to fully overlap the MEME-predicted palindromic binding sequence. Moreover, RccR binding to the genomic fragments tested occurs in the core promoter regions, encompassing the verified transcriptional start sites on p-*rccR* and p-*aceE*.

To further characterize RccR-DNA interactions, we carried out hydroxyl-radical footprinting assays (OH-FP). In this technique, DNaseI is substituted by radical ions as cutting agents to obtain a higher resolution (S4A Fig). For all three tested promoter probes, RccR binding resulted in a pattern of short protected tracts of 3/4 nucleotides in length, separated by non-protected regions. As before, nucleotides protected in the OH-FP experiment were mapped onto the promoter sequence (S4B Fig). Intriguingly, for all the tested binding sites, the nucleotides protected in OH-FP belong to the spacer that separates the conserved repeats of the consensus binding motif, or falls immediately upstream/downstream of the conserved repeats (S4B Fig). Regions protected in hydroxyl-radical footprinting experiments reflect the minor accessibility of radical ions to the DNA minor groove, and for this reason these protected regions do not necessarily represent the portions of the probe directly contacted by the protein. Considering that RccR footprint regions surround the inverted repeats, these data are consistent with RccR interaction with the conserved repeats in the DNA major groove, narrowing the adjacent minor grooves and protecting these sequences *in vitro* from hydroxyradical digestion. Additional binding experiments are required to fully characterise the RccR-DNA complex. Nonetheless, these data reinforce the crucial role of the conserved sequences in mediating specific RccR binding.

The RccR binding pattern on *aceE* is particularly complex (S4A Fig, lanes 9 to 12): there are 4 short protected stretches close to the conserved sequences (black boxes in S4A Fig and black dots in S4B Fig), and 2 additional protected tracts in the DNA region that separates the two binding sites (grey boxes in S4B Fig and grey dots in S4B Fig). Because this is a probe harbouring two separate operators bound to RccR, this suggests that the DNA fragment may undergo strong bending or a similar perturbation to enable RccR binding. The existence of two different RccR consensus binding sequences could explain why the pattern of regulation for *aceE* and *rccR* in different carbon sources differs markedly from the rest of the RccR regulon.

### RccR binds KDPG

As a member of the RpiR protein family, RccR contain an SIS domain for binding an effector-sugar. The high sequence homology between HexR and RccR and the conserved amino acids residues in the SIS domains, suggest that the RccR hypothetical effector could be a phosphorylated sugar, more likely an intermediate of the central carbon metabolic pathway controlled by both HexR and RccR. In order to identify the RccR ligand we performed SPR experiments, using the ReDCaT system, that allowed us to analyse the RccR-His binding on the *aceE*, *aceA* and *rccR* consensus sequences immobilised on the ReDCaT Chip, in presence of specific metabolic intermediates.

**Fig 9:**
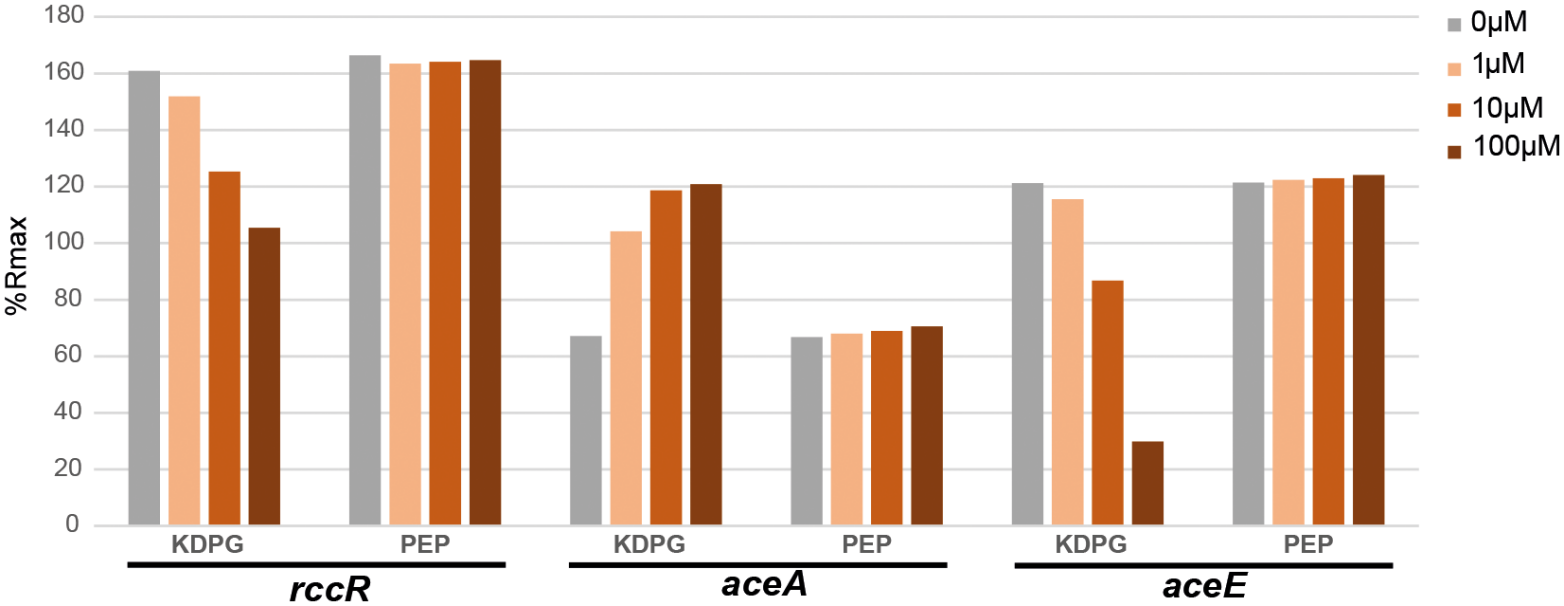
Screening for the RccR effector. **9A**: Percentage of normalized response (%Rmax) for RccR binding to the *rccR*, **9B**: *aceA* and **9C**: *aceE* consensus sequences in the presence of KDPG (effector) and PEP (negative control) at different concentrations (1-10-100 μM).

No response was seen for any of the tested sugars (S5 Fig, Fig 9), with the exception of the well-characterised HexR ligand KDPG (Fig 9). KDPG addition decreased the affinity of RccR-His for both *aceE* and *rccR* consensus sequences in the μM concentration range. Conversely, RccR binding affinity for the *aceA* (28bp) binding site increased in-line with a rising KDPG concentration (Fig 9). As such, it appears that KDPG is the specific RccR effector, and is responsible for the induction of the *aceE*/*F* operon and the conversion of pyruvate into acetyl-CoA. In the presence of KDPG, *rccR* auto-repression is reduced by a modest amount, in line with a shift from the 15 bp binding site to the 28 bp consensus on KDPG binding. Partial release of *rccR* repression, alongside increased binding affinity for the 28 bp sites upstream of the target genes, both serve to repress glyoxylate shunt/gluconeogenesis activity when KDPG levels are high.

### The RccR regulon is widespread and well-conserved

To assess the importance of RccR regulation in other bacterial species, a BLAST analysis with the SBW25 *rccR* gene sequence was conducted on 1502 publically-available bacterial genomes. Because of the high sequence identity shared by *rccR* and *hexR*, we performed a reciprocal BLASTp screen to discard false positive hits (representing *hexR* or other *rpiR* family members). This second step allowed us to confidently identify *rccR* homologs in numerous pathogenic and non-pathogenic *Pseudomonas* spp. and in dozens of additional bacterial genera (S1 Table). We next extended our analysis to examine the conservation of the RccR regulon throughout the *Pseudomonas* genus. A search of all publically-available *Pseudomonas* genomes was conducted using the consensus 28 bp and 15 bp pseudo-palindromic binding sequences, with stringent parameters to avoid false-positives (S2 Table). Multiple occurrences of the RccR consensus binding sequences were identified in almost every species in the genus. In every case, these binding sites were located upstream of genes found in the SBW25 RccR regulon, supporting a widespread and highly conserved role for RccR in the control of bacterial carbon metabolism and glyoxylate shunt regulation.

Given the widespread conservation of *rccR* and its importance for carbon utilisation and growth in *P*. *fluorescens*, we examined the similarity of the SBW25 regulatory network with that of the opportunistic human pathogen *P*. *aeruginosa* PAO1. The *P*. *aeruginosa* PA01 genome encodes a close homolog of *rccR* alongside most of the confirmed RccR gene targets identified in SBW25 (S3 Table). The only significant variation between the two regulons is the absence of a *PFLU2154* homolog in PA01. Manual examination of the upstream regions of the conserved genes in PA01 confirmed the presence of predicted RccR binding sequences in every case. Moreover, qRT-PCR expression analysis of the *rccR* gene targets in PA01, for cells grown in glycerol, pyruvate and acetate as the sole carbon source confirmed that RccR controls glyoxylate shunt and gluconeogenesis gene expression in *P*. *aeruginosa* in a similar fashion to *P*. *fluorescens* (S6 Fig).

## Discussion

In this study we identify and characterise a novel transcriptional regulator in *Pseudomonas* that regulates the expression of primary metabolic pathway genes in response to carbon source availability in the surrounding environment. The *rccR* gene was identified in a screen for up-regulated *loci* during *P*. *fluorescens* plant interaction [3], and encodes an RpiR family protein with a remarkably high amino acid sequence identity to the glycolysis regulator HexR. Members of the RpiR protein family contain an N-terminal helix-turn-helix DNA binding domain and a C-terminal ‘SIS’ sugar isomerase domain. The predicted structures of RccR and HexR are highly similar, with the important residues for HexR DNA and ligand binding [14] conserved in RccR. This high degree of structural similarity initially suggested that RccR and HexR may share a common role in the regulation of carbon metabolism, possibly as competitors for a shared binding site. Thus, we decided to examine HexR alongside RccR in *P*. *fluorescens* SBW25, and to determine the roles of both regulators during bacteria-plant interactions.

Both Δ*rccR* and Δ*hexR* SBW25 mutants displayed significantly compromised ability to colonize the rhizosphere of wheat seedlings, suggesting that both regulators contribute to efficient bacterial growth in the root environment, presumably via transcriptional control of carbon metabolism in response to the available plant root exudates. However, while both regulators are similarly important for efficient root colonisation, the *rccR/hexR* mutant strains show markedly different growth effects in minimal defined media. HexR is required for efficient SBW25 growth on pyruvate or acetate or succinate as the sole carbon source. *P*. *putida* HexR functions as a transcriptional repressor of genes involved in the glucose phosphorylative and Entner-Doudoroff pathways [14]. When bacteria grow on two/three-carbon sugars (or their precursors), HexR represses genes required for glucose transport and the initial stages of the Entner-Doudoroff pathway. Conversely, when glucose is available as a carbon source, these HexR gene targets are expressed. In the presence of glucose, the metabolic intermediate KDPG is produced and binds to the SIS domain of HexR. This leads to a release of DNA binding and the consequent transcriptional activation of the HexR regulon [14]. Based on our expression analysis of the Δ*hexR* mutant grown in different media conditions, *P*. *fluorescens* HexR clearly plays a similar role in regulating glucose metabolism as in other *Pseudomonas* spp. [14, 31]

**Fig 10:**
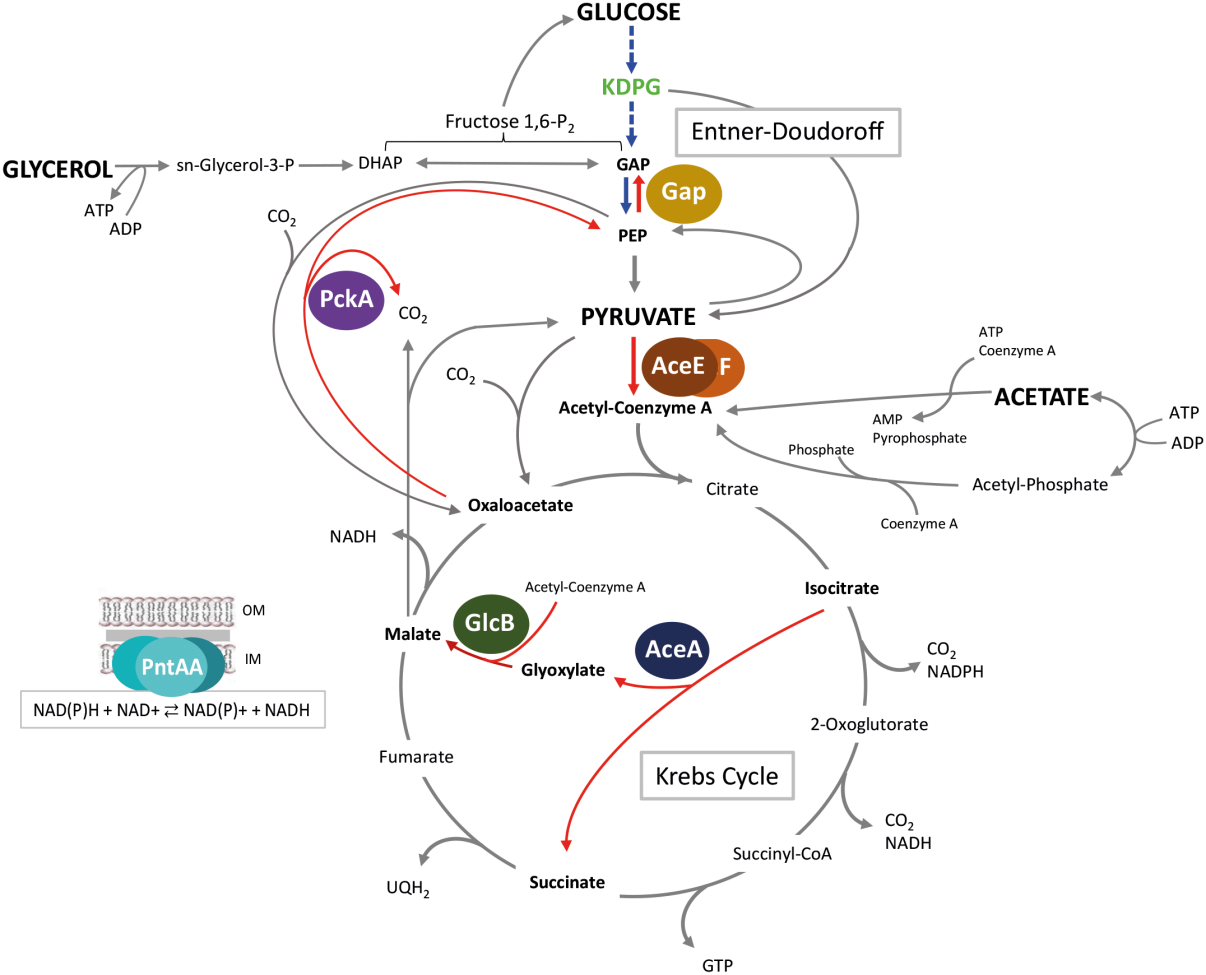
A model for RccR regulation of primary carbon metabolism. The figure shows a schematic representation of the metabolic pathways of glucose, glycerol, pyruvate and acetate through the Krebs cycle and the glyoxylate shunt. The protein products of the RccR gene targets are shown: PntAA/PFLU0112/B are subunits of the NAD(P) transhydrogenase membrane protein complex; PckA: phosphoenolpyruvate carboxykinase; AceE/F: pyruvate dehydrogenase subunits; Gap: glyceraldehyde-3-phosphate dehydrogenase; AceA: isocitrate lyase; GlcB: malate synthase G. RccR-regulated carbon transitions are marked in red. HexR-regulated carbon transitions are marked in blue.

Conversely, compromised growth of the Δ*rccR* mutant on glucose or glycerol suggests that RccR may work to produce an efficient metabolic response to an entirely different set of carbon sources. To understand the regulatory role of RccR in SBW25 we performed a ChIP-seq experiment that identified eight RccR binding sites, and a corresponding set of RccR gene targets. Firstly, RccR modulates its own expression, suppressing *rccR* gene expression in every condition tested, with the strongest repression seen for cells grown on acetate. Four of the RccR regulon genes; *aceA*, *glcB*, *pntAA*, and *pckA* are typically upregulated when bacteria grow in the presence of carbon sources that directly enter primary metabolism via acetyl-CoA, i.e. acetate, acetoacetate and fatty acids [33]. In agreement with this, our qRT-PCR analysis showed that these four genes are strongly repressed by RccR in glycerol and to a lesser extent in pyruvate, but not when acetate is the sole available carbon source. Isocitrate lyase (*aceA*) and malate synthase G (*glcB*) comprise the Glyoxylate shunt pathway, whose activation during growth on two-carbon molecules enables the replenishment of metabolic intermediates during Krebs cycle operation (Fig 10). The NADH/NAD+ cofactor pair plays a major role in microbial catabolism and it is crucially important for continued cell growth that NAD(P)H be oxidized to NAD(P)+ and a redox balance be achieved [34, 35].The *pntAA/0112/B* operon encodes a 3-subunit transhydrogenase enzyme that catalyses the reaction NADPH + NAD^+^ ⇄ NADP^+^ + NADH, based on the intracellular NADPH/NAD^+^ ratio. Finally, phosphoenolpyruvate carboxykinase (*pckA*) is involved in the first step of the gluconeogenesis pathway, a necessary anabolic pathway for bacterial growth on acetate and other two-carbon sources [36] (Fig 10).

Of the remaining RccR gene targets, two are suppressed during growth on glucose, glycerol or pyruvate, but less so on acetate. The *gap* gene encodes for glyceraldehyde-3-phosphate dehydrogenase (GAPDH). Interestingly, RccR and HexR appear to regulate the expression of two different isoenzymes, *gap* (PFLU1566) and *gap-1* (PFLU4965) working at the same metabolic step. However, these two isoenzymes have different roles in gluconeogenesis and hexose catabolism respectively, according to the relative levels of their preferred substrates (NAD^+^ and NADP^+^)[37]. *PFLU2154* encodes a hypothetical protein of unknown structure and function, and is particularly strongly repressed in glycerol medium. This gene is apparently not essential, as it can be deleted in SBW25 and is not present in every *Pseudomonas* species. The defective growth of the Δ*PFLU2154* mutant strain in acetate medium suggests a role for this protein in either two-carbon source metabolism or gluconeogenesis, consistent with our expression data for the RccR regulon.

The final RccR gene target is the *aceE/F* operon, which presents an entirely different regulatory pattern to the other members of the RccR regulon. These genes, encoding two subunits of a pyruvate dehydrogenase, are expressed in the presence of glucose, glycerol and pyruvate but are strongly repressed in acetate. This makes sense in the context of a switch from glycolysis to two-carbon metabolism and gluconeogenesis. AceEF suppression effectively prevents the further metabolism of pyruvate to acetyl-CoA, enabling the accumulation of carbon above this stage of glycolysis by PckA (Fig 10). Clearly, when grown on acetate as the sole carbon source, RccR enables both the repression and de-repression of different gene targets to produce an integrated regulatory response.

MEME analysis of the sequences surrounding each of the 8 RccR binding sites presents a first step towards understanding this intricate regulatory system. A pseudo-palindromic, 28 bp consensus sequence was identified in the upstream regions of the seven glycerol/pyruvate suppressed RccR targets, while two copies of a shorter, 15 bp pseudo palindrome were found upstream of the *aceE/F* operon. The consensus sequence of these sites is the same (TGTAGT/ACTACA), with the only difference the length of the internal linker. Subsequent SPR and Footprinting analyses confirmed that RccR binds to both of these consensus sequences, albeit with markedly different binding affinities. The presence of two distinct but related binding sites upstream of the RccR targets suggests that RccR exerts discrete regulatory effects on different genes in its operon through distinct DNA binding mechanisms. The 28 bp consensus sequence found upstream of *rccR* is particularly interesting in this context, as it contains the 15 bp consensus sequence within it. The presence of this double binding site upstream of the *rccR* gene could explain the constant repression observed for *rccR* in both acetate and glycerol/pyruvate media.

Overlaying the RccR binding sites onto the six 5’ RACE-mapped promoter regions (S3 Fig) adds an additional layer of complexity to the RccR regulon. RccR binding overlaps (or is very close to) the +1 sites of *pckA*, *aceE*, *glcB* and *rccR*, implying a role for RccR in inhibition of promoter escape or inhibition of open complex formation on these promoters [38]. On the other hand, the RccR binding sites on the *aceA* and *PFLU2154* promoters are located 40/41 bases upstream of the +1 site, suggesting a distinct repression mechanism applies for *aceA* and *PFLU2154*. This would also be consistent with the much stronger conditional repression (relative to *pckA* and *glcB*) seen for these genes in our q-PCR experiments.

Informed by the structural similarity between HexR and RccR, we confirmed the ED pathway intermediate KDPG as the sugar ligand for RccR. Identifying this RccR effector was the breakthrough that allowed us to interpret how the intricate HexR/RccR regulon controls primary carbon metabolism in *Pseudomonas*. In the absence of ligand binding, RccR-His has a far higher affinity for DNA sequences containing the 15bp consensus binding site than for the 28bp site. As repression of the *aceEF* operon only occurs in acetate-containing media, the affinity for this consensus sequence must decrease in conditions where pyruvate needs to be converted into acetyl-CoA. As predicted, KDPG binding to RccR markedly decreases binding affinity to the 15bp consensus, consistent with release of *aceEF* repression. Similarly, *rccR* expression is itself slightly de-repressed in the presence of the ligand. Conversely, KDPG binding RccR enhances binding to the lower-affinity 28bp binding sites upstream of *aceA*, *pckA* and other glyoxylate shunt/gluconeogenesis genes. Combined with the mildly increased expression of the RccR repressor itself, this results in strong inhibition of gene transcription from these loci when KDPG is present. This regulatory mechanism may help to explain the intermediate growth defect seen for the *hexR/rccR* double mutant grown on acetate media (Fig 2F). In the other tested carbon conditions, the double mutant phenocopies the defective single mutant—i.e. the loss of transcriptional regulation of either the glyoxylate shunt or the ED pathway leads to inefficient metabolism and a growth defect. Most of the time, the additional loss of the non-repressing regulator is phenotypically neutral. In acetate, we propose that uncontrolled KDPG production in the *hexR* knockout leads to RccR inhibition and the loss of glyoxylate shunt function. Recovery of glyoxylate shunt activity in the double mutant allows the cells to use the available 2C carbon source, leading to a partial recovery of growth rate. By combining the activities of RccR and HexR, *P*. *fluorescens* controls multiple stages of primary carbon metabolism, from the glucose phosphorylative and Entner-Doudoroff pathways, to pyruvate catabolism, glyoxylate shunt and gluconeogenesis, by monitoring the concentration of a single metabolic intermediate, KDPG.

Why the 15 and 28bp binding sites show such different affinities, and opposing responses to RccR ligand binding is currently unclear, but may be related to the length of the linker regions and the dimerization state of the transcriptional regulator. Because the length of a full DNA turn is 10.5 nucleotides, two RccR monomers binding to the shorter consensus can easily interact with each other on the same DNA face. Conversely, such interactions are likely to be more difficult on the 28bp consensus sequence, where our data suggest that DNA binding is required to enable RccR homodimer interaction. If KDPG binding changes the conformation of the RccR dimer, this may simultaneously stabilise the more flexible long-range complex and inhibit binding to the higher-affinity but less flexible 15bp site. A structural analysis of the RccR-DNA bound complex would be helpful for clarify the true mechanism of RccR function.

Our analysis shows that the RccR regulator is conserved across multiple bacterial genera, and in every tested *Pseudomonas* species, supporting the importance of this transcription factor in the control of carbon metabolism. Given the strong similarity between RccR and HexR, it is reasonable to assume that these proteins are the product of gene duplication at some point in the past. Further evidence in support of this comes from the *Shewanella oneidensis* HexR homolog, which is proposed to control the ED and glucose phosphorylative pathways as well as the glyoxylate shunt and gluconeogenesis [39]. In *Pseudomonas* this role has clearly been subdivided between the “twin” proteins HexR and RccR.

The RccR/HexR pathway enables *P*. *fluorescens* to dynamically fine-tune its metabolic pathways to quickly respond to carbon source availability. By using two transcriptional regulators with three different DNA target sequences to sense the concentration of a single key metabolic intermediate, RccR/HexR represents an elegant mechanism to enable environmental adaptation.

## Materials and methods

### Strains, growth conditions and molecular biology procedures

*P*. *fluorescens* strains were grown at 28°C, while *P*. *aeruginos*a and *E*. *coli* were grown at 37°C in lysogenic broth (LB), solidified with 1.5% agar where appropriate. For growth experiments, bacteria were grown in M9 minimal media with single carbon sources added to a final concentration of 0.4% w/v, unless otherwise stated. Kanamycin (Kc) was used at 50 μg/ml, Gentamicin (Gm) was used at 12.5 μg/ml, Tetracycline (Tet) was used at 10 μg/ml for *E*. *coli*, 12.5 for *P*. *fluorescens* and 100 μg/ml for *P*. *aeruginosa*, while Carbenicillin (Cb) was used at 100 μg/ml. X-Gal was used at 40 μg/ml and IPTG at 100 μg/ml. All bacteria strains and plasmids used in this study are listed in S4 Table.

Common molecular biology methods including plasmid DNA extraction, transformation, cloning, restriction digests, electrophoresis, purification of DNA fragments and sequencing were carried out according to standard molecular biology techniques as described previously [40]. PCR reactions were performed using GoTaq or Phusion DNA polymerase as appropriate. All oligonucleotides used in this study are listed in S5 Table and have been designed mainly on the basis of the *P*. *fluorescens* SBW25 (GenBank NC_012660.1) and *P*. *aeruginosa* PAO1 (GenBank NC_002516) genome sequences.

### Gene deletions

*P*. *fluorescens* and *P*. *aeruginosa* deletion mutants were constructed via an adaptation of the protocol described elsewhere [41]. Up- and downstream flanking regions to the target genes were amplified by PCR using primers: 1–2, 3–4 and 11–12, 13–14 for *rccR* deletion in SBW25 and PAO1 respectively; 5–6, 7–8 and for *hexR* deletion in SBW25; 17–18, 19–20 for *PFLU2154* deletion. The products in each case were ligated into pTS-1 between *Xho*I-*Bam*HI. The resulting vectors were transformed into the target strain, and single crossovers were selected on Tet and re-streaked for single colonies. Cultures from single crossovers were grown overnight in 50 ml LB medium, then a dilution series of the overnight culture was plated onto LB plates containing 10% sucrose to enable counter-selection. Individual *P*. *fluorescens* colonies were patched onto LB plates ± Tet, with Tet-sensitive colonies tested for gene deletion by colony PCR using external primers.

### *In silico* analysis

HexR and RccR sequence comparisons were carried out using the NCBI BLAST online tools (http://blast.ncbi.nlm.nih.gov/Blast.cgi). The model protein structure of SBW25 RccR was constructed using HHPred (Homology detection & structure prediction), followed by HMM-HMM comparison and MODELLER. The model generated was based on 4 published crystal structures (PDB file names: 3sho, 2o3f, 4ivn, and 3iwf). To search for *rccR* by reciprocal BLAST using BLASTp, we randomly selected one species from each of the annotated bacterial genera available in the EBI collection of bacterial genomes (https://www.ebi.ac.uk/genomes/bacteria.details.txt). This resulted in 1502 bacterial genomes being searched, with the results of the search shown in S1 Table. The sample pool contained one randomly selected representative of each publically available, annotated bacterial species, from a total available collection of 3794 genomes at NCBI. RccR transcriptional binding sites were identified by analysing the sequences surrounding the RccR ChIP-seq peaks using MEME (Multiple EM for Motif Elicitation) (http://meme.nbcr.net) [42], with additional manual analysis of each region conducted as discussed in the results. All *Pseudomonas* genomes in the EBI collection of bacterial genomes were searched with the RccR binding site consensus by doing a simple string search (using Perl) allowing for 1, 2 or 3 mismatches over the entire length of the consensus sequence. The results are shown in S2 Table.

### Root colonisation assays

Paragon wheat seeds were sterilized with 70% ethanol and 5% hypochlorite, washed and germinated on sterile 0.8% MS agar for 72 h in the dark. Seedlings were then transferred into sterile 50 ml tubes containing medium grain vermiculite and rooting solution (1 mM CaCl_2_.2H_2_O, 100 μM KCl, 800 μM MgSO_4_, 10 μM FeEDTA, 35 μM H_3_BO_3_, 9 μM MnCl_2_.4H_2_O, 0.8 μM ZnCl_2_, 0.5 μM Na_2_MoO_4_.2H_2_O, 0.3 μM CuSO_4_.5H_2_O, 6 mM KNO_3_, 18.4 mM KH_2_PO_4_, and 20 mM Na_2_HPO_4_), and transferred to a controlled environment room (25°C, 16h light cycle). WT-*lacZ* and mutant SBW25 strains were grown overnight in M9 0.4% pyruvate media, then serially diluted in phosphate buffer. 1 x 10^3^ CFU of mutant and WT-*lacZ* bacteria/plant were used to inoculate seven day-old wheat seedlings. Plants were grown for a further seven days, after which shoots were removed and 20 ml PBS was added to each tube and vortexed thoroughly to resuspend bacteria. A dilution series was plated onto XGal + IPTG + Cb plates and WT-*lacZ*/mutant colonies distinguished by blue/white selection. Assays were conducted for 10 plants/mutant, repeated at least twice independently, and statistical significance assessed using Mann-Whitney tests [43].

### Growth assays

Bacterial growth was monitored in a microplate spectrophotometer (BioTek Instruments) with a minimum of 3 experimental replicates/sample. Wells (of a 96-well plate) contained 150 μL of the appropriate growth medium. Growth was initiated by the addition of 5μL of cell culture with an OD_600_ = 0.01. Plates were covered with adhesive sealing sheets and incubated statically at 28°C or at 37°C for *P*. *fluorescens* and *P*. *aeruginosa* respectively. Each experiment was repeated at least twice independently.

### ChIP-seq assay

Chromatin Immunoprecipitation (ChIP) assays were performed as described elsewhere [44], with the following modifications. After bacterial growth, formaldehyde (1%) was added to the cultures and incubated at room temperature for 20 min, before the reaction was quenched with glycine (125 mM) for 5 min. Cells were collected and washed with cold PBS four times. The cells were lysed in 1 ml of lysis buffer solution (10 mM Tris-HCl pH8, 50 mM NaCl, 4 mg/ml lysozyme, 1x protease inhibitor). Cell extracts were then sonicated to fragment the DNA to an average size of 500bp. 50 μl of the extract was removed for total DNA preparation. For immunoprecipitation of RccR cross-linked DNA, a portion of the extract (1 ml) was immunoprecipitated with 10 μl of polyclonal anti-RccR antibody at 4°C for 4h. After incubation with ProteinA affinity gel (Sigma) for 1h at 4°C, the beads were washed twice with IP buffer (100 mM Tris-HCl pH 8.0, 250 mM NaCl, 0.5% Triton X-100, 0,1% SDS, 1x protease inhibitor), and finally with TE buffer. The immunoprecipitated material was eluted with 100 μl of elution buffer (50 mM Tris-Cl pH 7.6, 10 mM EDTA, 1% SDS). Cross-linking of immunoprecipitated and total DNA was reversed by incubation at 65°C overnight. After Proteinase K (Roche) treatment, the immunoprecipitate and total DNA samples were extracted using phenol-chloroform and purified using a QiaQuick kit (Qiagen). Illumina TruSeq ChIP-seq libraries were produced from these samples and size selected to ~200–300 bp., then sequenced on a single Illumina HiSeq 2000/2500 lane in High Output mode, using 100 bp single-end reads.

### ChIP-seq data analysis

The reads in the fastq files received from the sequencing contractor were aligned to the *Pseudomonas fluorescens* SBW25 genome using the bowtie2 software, which resulted in one SAM (.sam) file for each fastq file. All further operations described below were carried out using a combination of Perl scripts dependent on the BioPerl toolkit and R scripts. From each SAM file, coverage at (i.e. number of reads mapping to) each nucleotide position of the *Pseudomonas fluorescens* SBW25 genome was calculated and the output was saved in files, referred to here as coverage files. For each coverage file, a local enrichment was calculated in a moving window of 51 nucleotides (nt) moving in steps of 25 nucleotides as (the sum of coverage at each nucleotide position in the 51-nt window) divided by (the sum of coverage at each nucleotide position in a 4,001-nucleotide window centred around the 51-nucleotide window). This results in an enrichment ratio value for every 25 nucleotides along the genome. All nucleotide positions where the enrichment ratio was less than 1.5 were removed, then a negative binomial distribution was fitted to the data using the fitdistr function of the MASS package in R. Thus, we arrived at the size and mu parameters of the binomial distribution. The values of size and mu parameters resulting from the fitting of the binomial distribution were then used to calculate *p*-values for each enrichment ratio using the pnbinom function of R. Finally, the *p*-values were adjusted for multiple testing by using the p.adjust function of R using the Benjamini and Hochberg method. This resulted in tables with three columns: Genomic position, Enrichment ratio and Adjusted P-value. Information on genes to the left and right of each genomic position was added to these tables using gene coordinates from the SBW25 GenBank file. The ChIP-seq data has been deposited in the ArrayExpress database (accession number E-MTAB-5745).

### β-galactosidase assay

β-galactosidase assays were performed as previously described [45]. Cells were grown to OD_600_ 0.5–0.6 in M9 media supplemented with glucose/glycerol/pyruvate/acetate, then permeabilized with sodium dodecyl sulphate and chloroform. β-galactosidase activity was assayed using o-nitrophenyl-b-D-galactopyranoside (ONPG) as a substrate, and is reported as μmol o-nitrophenol/min/mg cellular protein. The results obtained were normalized to the UM recorded for WT strains carrying the plasmids pGm-p-*rccR* or pGM-p-*hexR* (S4 Table). Experiments showing the expression of *lacZ* fusions in single-value data are the average of at least four independent measurements.

### RNA extraction

Total RNA was extracted from 50ml cultures of SBW25/PA01 WT and Δ*rccR*/Δ*hexR* mutants grown in M9 minimal media to OD_600_ = 0.6, or after Media exchange where appropriate (below). 30 ml of 60% RNAlater (in PBS) was added to each tube, and sealed tubes were vortexed and centrifuged for 10 min at 4°C. The pellets were resuspended in 1x PBS + chilled β-mercaptoethanol RT solution, and lysed by mechanical disruption. Finally, RNA was purified from the lysate by column capture using an RNeasy Mini Kit (Qiagen). Purified RNA was subjected to an additional DNase treatment (Turbo^™^ DNase, Ambion). RNA quantification was performed with an ND-1000 Spectrophotometer.

### Media exchange

WT and mutant strains were grown in LB overnight at 28°C. The next day, the overnight cultures were diluted in M9 pyruvate and grown until OD_600_ = 0.3. Cells were then pelleted by centrifugation and resuspended in an equal volume of Rooting Solution without any carbon sources (see Colonisation assay for composition). After 1 hour of incubation at 28°C, the cells were pelleted by centrifugation, then resuspended in 50 ml M9 supplemented with 0.4% glucose/ glycerol/ pyruvate/ acetate. Cultures were incubated for 30 min at 28°C, and RNA was extracted as described above.

### Quantitative Real time PCR (qRT-PCR)

cDNA synthesis was performed as previously described [4]. Real time PCR was performed using a 20 μl reaction mix containing 1 μl cDNA. Primers from number 29 to 75 listed in S5 Table were used for the tested gene expressions. At least three wells were run for each sample. The amount of gene transcript in each case was analysed by Absolute or Relative studies (2^−ΔΔCt^ method) [46, 47]. Absolute quantification was used to determine the number of copies of gene targets (*rccR* or *hexR*) in our WT strains without reference to other samples. For this analysis a standard curve was constructed (in duplicate) using SBW25 chromosomal DNA for the quantification of transcripts. All gene quantifications were normalized to the levels of the endogenous gene *rpoD*. Relative quantification was used to compare the abundance of *rccR* (or *hexR*) gene target mRNAs in equivalent WT and *ΔrccR* (or *ΔhexR*) samples. The amount of each gene transcript was normalized to the WT reference sample. For the 2^−ΔΔCt^ method, results were presented as n-fold increase relative to the reference sample. The ΔCt-values were examined using the Student's t test to determine whether datasets for relative gene expression were significantly different from those in a chosen calibrator. Primers for the aforementioned transcripts and for the *rpoD* transcript, used as an endogenous control, were experimentally validated for suitability to the 2^−ΔΔCt^ method, and are listed in S5 Table. Melting curve analysis was used to confirm the production of a specific single product from each primer pair. Each experiment was repeated at least twice independently.

### Protein purification

The *rccR* gene was cloned into pET42b to give the expression vector pET42b-*rccR*. This construct was transformed into *E*.*coli* strain BL21 (DE3). 25 ml of an overnight culture of BL21pLys pET42b-*rccR* were used to inoculate 4L of LB supplemented with Kc. Cells were grown at 28°C to an OD_600_ of 0.4, then 1 mM isopropyl β-D-thiogalactopyranoside was added to induce *rccR* expression. After 2h shaking, the cells were harvested by centrifugation, the pellet was resuspended in 40ml Equilibration buffer (20mM Hepes, 250mM NaCl, 10mM MgCl_2_, 2.5% glycerol, pH 6.8) and lysed by sonication. An additional centrifugation step removed cell debris, then the supernatant was applied to 1 ml nickel-charged HisTrap chelating column attached to an Äkta FPLC (GEHealthcare). The column was washed with Equilibration buffer, while the protein was eluted with a linear gradient of imidazole to 1M in the Elution Buffer (20mM Hepes, 150mM NaCl, pH7.4). The fractions containing RccR-His were pooled and applied to 5ml heparin column. The column was washed with Buffer A (20mM Hepes, 50mM NaTiocianate, pH7.4), while the elution was conducted using Buffer B (20mM Hepes 1M NaCl, pH7.4). The fractions containing RccR were then combined. The C-terminal His tag was not removed from the purified protein for any of the subsequent experiments.

### Transcriptional start site identification

Transcriptional start site identification for the RccR gene targets was carried out using the Invitrogen kit 5’ RACE System for Rapid Amplification of cDNA Ends, Version 2.0. Total RNA were purified from *P*. *fluorescens* as above, then mRNA enrichment was carried out using a specific primer GSP1 for the RNA target. After reverse transcription to cDNA using SuperScriptII RT, RNA was degraded from the samples using an RNase mix. The cDNA was purified using GlassMAX Spin Cartridge, then tailed with dCTP and TdT. After the tailing reaction, a second PCR reaction using the Abridged Anchor Primer (AAP) and a GSP2 nested primer was performed. Finally, a third PCR to re-amplify the previous PCR product was conducted, using the AUAP primer (that recognises the AAP region), and a GSP3 nested primer. The final PCR product was sequenced with Big Dye 3.1 by Eurofins.

### Surface plasmon resonance

All the SPR experiments described here were performed using a GE Healthcare Biacore T200 instrument. All measurements were recorded at 25°C using the ReDCaT system described in [32], with a single SensorChip SA (GE Healthcare) having four flow cells each containing SA pre-immobilized to a carboxymethylated dextran matrix. DNA samples were purchased from Eurofins Genomics as desalted single-stranded (ss) oligomers at 100 μM concentration in water, while the RccR-His protein was purified as described above. These experiments were performed based on knowledge of the predicted RccR consensus sequences, so the DNA fragments were designed *ad hoc*, with a length of about 30 nt each (S5 Table, 93/110). The DNA was prepared by taking 45 μl of the shorter strand and mixing with 55 μl of the longer strand. This was then heated to 95°C and cooled to room temperature. This gave a 45 μM stock which was then diluted to 1 μM in running buffer (10 mM Hepes pH 7.4, 300 mM NaCl, 3mM EDTA, 0.05% v/v tween20). For each experiment, all protein samples were diluted in running buffer. For all the SPR experiments the DNA at 1uM was captured at the start of each cycle by loading at 10μl/min on FC2. Interaction with RccR protein was measured by flowing the RccR protein over both FC1 and FC2 for 60s at a flow of 30μl/min. The chip was then washed to remove any protein still bound and the DNA by washing with 1 M NaCl, 50mM NaOH at the end of each cycle.

Initial screening experiments were run to measure RccR binding to each DNA sequence target. Concentrations of 1 μM and 0.1 μM of RccR protein were used. The amount of DNA captured and the binding response of RccR was measured and this was converted to the %R_max_ bound (percentage of the theoretical maximum response), assuming a single RccR dimer binding to a single immobilized ds DNA oligomer (S6 Table). The affinity of RccR for the *aceE*, *aceA* and *rccR* consensus sequences was then determined, The RccR protein stock (40 mM) was serially diluted in running buffer to give concentrations of 100, 50, 25, 12.5, 6.25, 3.125, 1.56, 0.78, 0.39 and 0.19 μM. Measurements were taken in triplicate for a range of protein concentrations spanning either side of the expected *K*_d_ using a multicycle kinetic approach.

For RccR ligand identification, binding experiments were run to measure the association of RccR with *aceE*, *aceA* and *rccR* consensus sequences in the presence of different carbon metabolites. Several samples (RccR—hypothetical ligand mixes) were prepared, so that RccR protein was diluted to 0.5 μM in running buffer containing the tested ligand at different concentrations (0-1-10-100-1000 μM) with a final composition matching the running buffer. The %Rmax bound, assuming a single RccR dimer binding to one immobilized ds DNA oligomer was calculated (S6 Table).

### DNase I footprinting

The regions of the *P*. *fluorescens* SBW25 genome containing the predicted RccR binding sites upstream of the coding sequences of *aceA*, *aceE* and *rccR* were PCR amplified with oligonucleotides 81–82, 83–84, 85–86, respectively, and the generated DNA fragments inserted into the plasmid pGEM-T-Easy (Promega). The DNA probes were 5’-end labelled and the assays carried out as previously described [48]. Briefly, 1 pmol of pGEM-T-Easy-P*aceA*, pGEM-T-Easy-P*aceE* and pGEM-T-Easy-P*rccR* were linearized by SpeI digestion, dephosphorylated with calf intestinal phosphatase and 5’-end labelled with T4 polynucleotide kinase in the presence of 2 pmol of [γ32-P]-ATP (6000 Ci/mmol; Perkin Elmer). Following *Nco*I digestion, the labelled DNA fragments were gel purified and resuspended in ddH2O. For footprinting assays, approximately 20 fmol of the labelled probes were incubated with purified RccR protein in 50 μL of 1X Footprinting Buffer (10 mM Tris-HCl pH 8.0; 50 mM NaCl; 10 mM KCl; 5 mM MgCl_2_; 1 mM DTT; 0.01% NP-40; 10% glycerol) containing 200 ng of sonicated salmon sperm DNA as a non-specific competitor, for 15 min at room temperature; afterwards, 0.04 units of DNase I (EMD Millipore), freshly diluted in Footprinting Buffer containing 5 mM CaCl_2_, were added to the reaction mixture, and the digestion was allowed to occur for 75 seconds at room temperature. After stopping the reactions, samples were phenol-chloroform extracted, ethanol precipitated and resuspended in Formamide Loading Buffer (95% formamide; 10 mM EDTA; 0.02% bromophenol blue; 0.02% xylene cyanol). Next, samples were denatured at 100°C for 5 min, separated on 8 M urea–6% polyacrylamide sequencing gels in TBE buffer and autoradiographed.

### Hydroxyl-radical footprinting

Genomic regions *P*. *fluorescens* SBW25 encompassing RccR binding sites on *aceE*, *aceA* and *rccR* promoters were PCR amplified with specific primers (87–88, 89–90, 91–92) and cloned into the plasmid pGEM-T-Easy. Then, DNA probes were 5'-end labelled (using *Bam*HI and *Xho*I for the sequential digestions) and purified as described above for DNase I footprintings. Hydroxyl-radical footprinting experiments were performed as previously described [49] with some modifications. Approximately 20 fmol of labelled probes were incubated with increasing concentrations of RccR protein in OH-Footprinting Buffer (10 mM Tris-HCl, pH 8.0; 50 mM NaCl; 10 mM KCl; 5 mM MgCl_2_; 0.1 mM DTT; 0.01% NP_4_0) for 15 minutes at room temperature, including 200 ng of sonicated salmon sperm DNA as a non-specific competitor in a final volume of 30 μL. The digestions of the labelled probes were performed using 2 μL each of the following solutions: 125 mM Fe (NH_4_)_2_(SO_4_)_2_-, 250 mM EDTA, 1% H_2_O_2_ and 100 mM DTT. After 2 minutes, for each sample the reaction was quenched by the addition of 25 μL of OH Stop Buffer (4% glycerol; 600 mM NaOAc, pH 5.2; 100 ng/μL sonicated salmon sperm DNA). Samples were phenol/chloroform extracted, ethanol precipitated and resuspended in 12 μL of Formamide Loading Buffer. Next, samples were denatured at 100°C for 5 minutes, separated on an 8M urea-8.5% polyacrylamide sequencing gel in TBE buffer and autoradiographed.

## Supporting information

S1 Fig **S1A-C : Expression of the divergent genes found in the RccR ChIP-seq experiment** was analysed by qRT-PCR in the Δ*rccR* mutant relative to WT SBW25 in glycerol media. These experiments show that only single transcriptional units are regulated by RccR in each case: **S1A**: PFLU0460, **S1B**: PFLU1566 and **S1C**: PFLU5623.
(TIF)

S2 Fig **S2A : Growth curves** for SBW25 and Δ*2154* strains in LB, **S2B**: in KB media, **S2C**: in M9 0.4% glucose, **S2D**: in M9 0.4% pyruvate, **S2E**: in M9 0.4% glycerol, **S2F**: in M9 0.4% acetate, **S2G**: in M9 0.4% malate, and **S2H**: in M9 0.4% succinate. The only significant difference in growth rate was seen between WT and Δ*rccR* in acetate (**S3F**). Experiments were repeated at least three times independently. **S2I: Rhizosphere colonisation competition assays.** The graph shows the ratio of SBW25 WT or Δ*2154* to WT-*lacZ* colony forming units (CFU) recovered from the rhizospheres of wheat plants seven days post-inoculation. Each dot represents CFU recovered from an individual plant. No significant differences in colonisation efficiency were seen between SBW25 and Δ*2154*. Experiments were repeated at least twice independently.
(TIF)

S3 Fig RccR binding target transcriptional start site identification. Schematic representation of *pckA*, *aceE*, *PFLU2154*, *aceA*, *glcB* and *rccR* mapping start sites. In red the RccR consensus sequences; in green the guanosines that are possible starting sites or belonging to the tagging tail.
(TIF)

S4 Fig Defining the RccR binding sites with hydroxyl-radical footprinting. **S4A**: hydroxyl-radical footprintings panel of RccR on *rccR*, *aceA* and *aceE* promoters. RccR-His protein concentrations used in this set of experiments: 0, 40, 80, 160 nM for *rccR* (lanes 1 to 4) and *aceE* (lanes 9 to 12); 0, 80, 160, 320 nM for *aceA* (lanes 5 to 8). Leftmost lane of each autoradiograph: Maxam and Gilbert G+A sequence reaction ladder. Symbols are detailed in the legend of Fig 8 Panel A (in the rightmost panel, grey boxes on the right depict protected tracts in the DNA region that separates the two principal binding sites). **S4B**: nucleotides protected in OH-FP were mapped on the respective promoter sequences. Black/grey dots indicate nucleotides protected, while protected regions in DNaseI footprintings (Fig 8A/8B) are included in an open dashed box and conserved pseudopalindromic sequences are highlighted in light grey. Bent arrows indicate the transcriptional start sites identified in this study (S3 Fig) and the first transcribed nucleotides are in bold.
(TIF)

S5 Fig SPR negative results of the tested hypothetical RccR ligand binding. (TIF)

S6 Fig **The RccR regulon in *Pseudomonas aeruginosa* S6A-C**: PA01 RccR gene target expression determined by qRT-PCR. Data are shown for PA01 *ΔrccR* relative to WT in **S6A**: glycerol media, **S6B**: pyruvate media, and **S7C**: in acetate media.
(TIF)

S1 Table *rccR* reciprocal BLASTp analysis. The *rccR* gene is conserved in 1502 genomes of different bacterial strains picked up randomly from a collection of 3794 bacterial genomes. The most significant data (p-value <0.05) are in a green box, where sequences overlapping > 70% (Hcov >0.7).
(XLSX)

S2 Table Conserved *rccR* binding sites into upstream regions of the homologous RccR gene targets in *Pseudomonas*. The 28 bp consensus sequences are grouped in different coloured boxes, relative to their targets. In yellow *pntAA*, in blue *gap*, in orange *pckA*, in green *glcB*, in light blue PFLU2154 hypothetical protein, in violet *aceA*, in grey *rccR*. The 15 bp consensus sequence relative to *aceE* is shown in the pink box.
(XLSX)

S3 Table RccR consensus sequences in the upstream region of RccR gene targets in *P*. *fluorescens* and *P*. *aeruginosa*. (DOCX)

S4 Table Strains and vectors. (DOCX)

S5 Table Primers. (DOCX)

S6 Table %Rmax calculation for the SPR experiments. (XLSX)
