# Supplementary figures and images for: One ligand, two regulators and three binding sites: How KDPG controls primary carbon metabolism in Pseudomonas
Source: PLoS Genet. 2017 Jun 28;13(6):e1006839. doi: 10.1371/journal.pgen.1006839 (PMC5489143; doi:10.1371/journal.pgen.1006839)

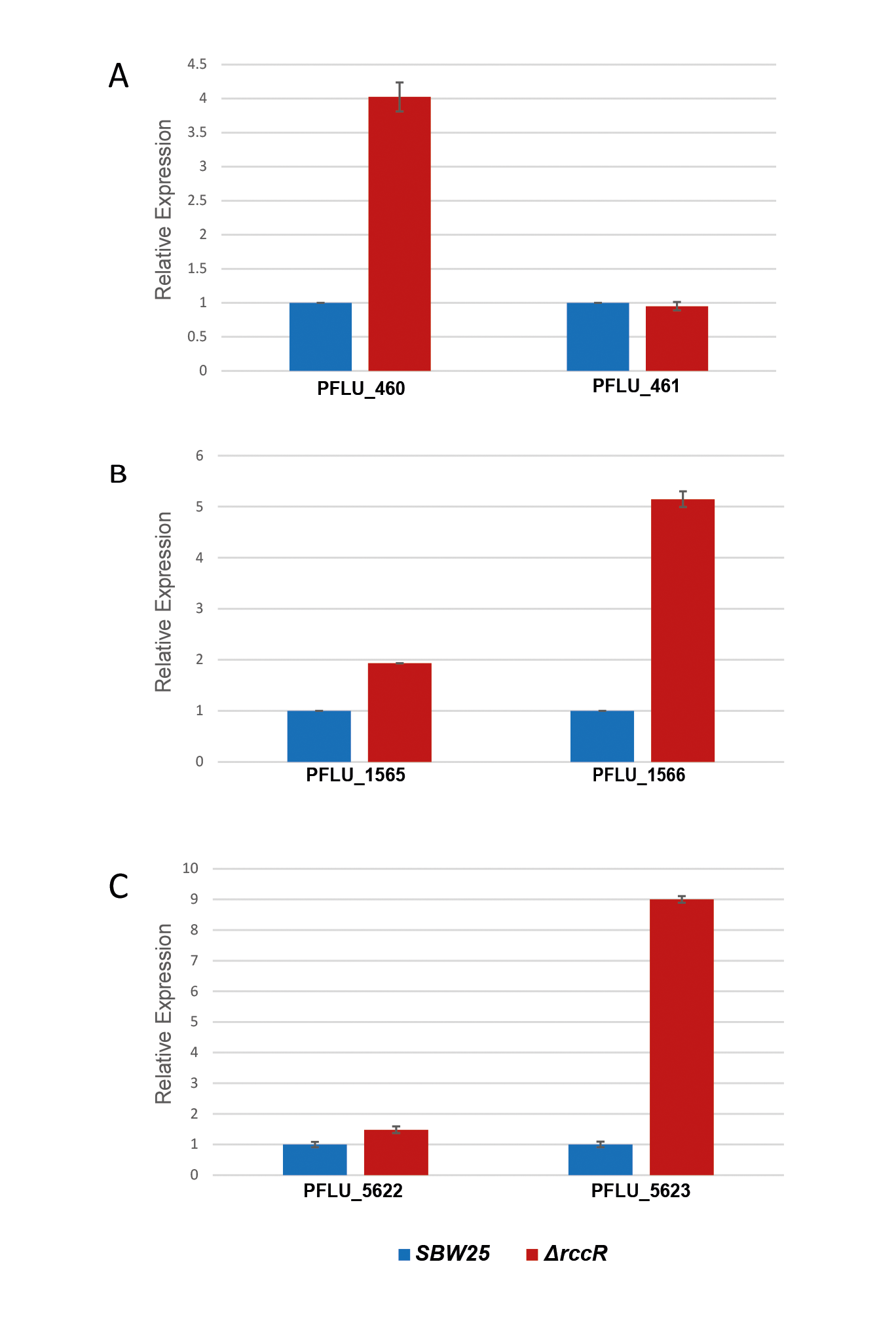

Supplement: S1 Fig — S1A-C : Expression of the divergent genes found in the RccR ChIP-seq experiment was analysed by qRT-PCR in the ΔrccR mutant relative to WT SBW25 in glycerol media. These experiments show that only single transcriptional units are regulated by RccR in each case: S1A: PFLU0460, S1B: PFLU1566 and S1C: PFLU5623. (TIF) [file pgen.1006839.s001.tif]

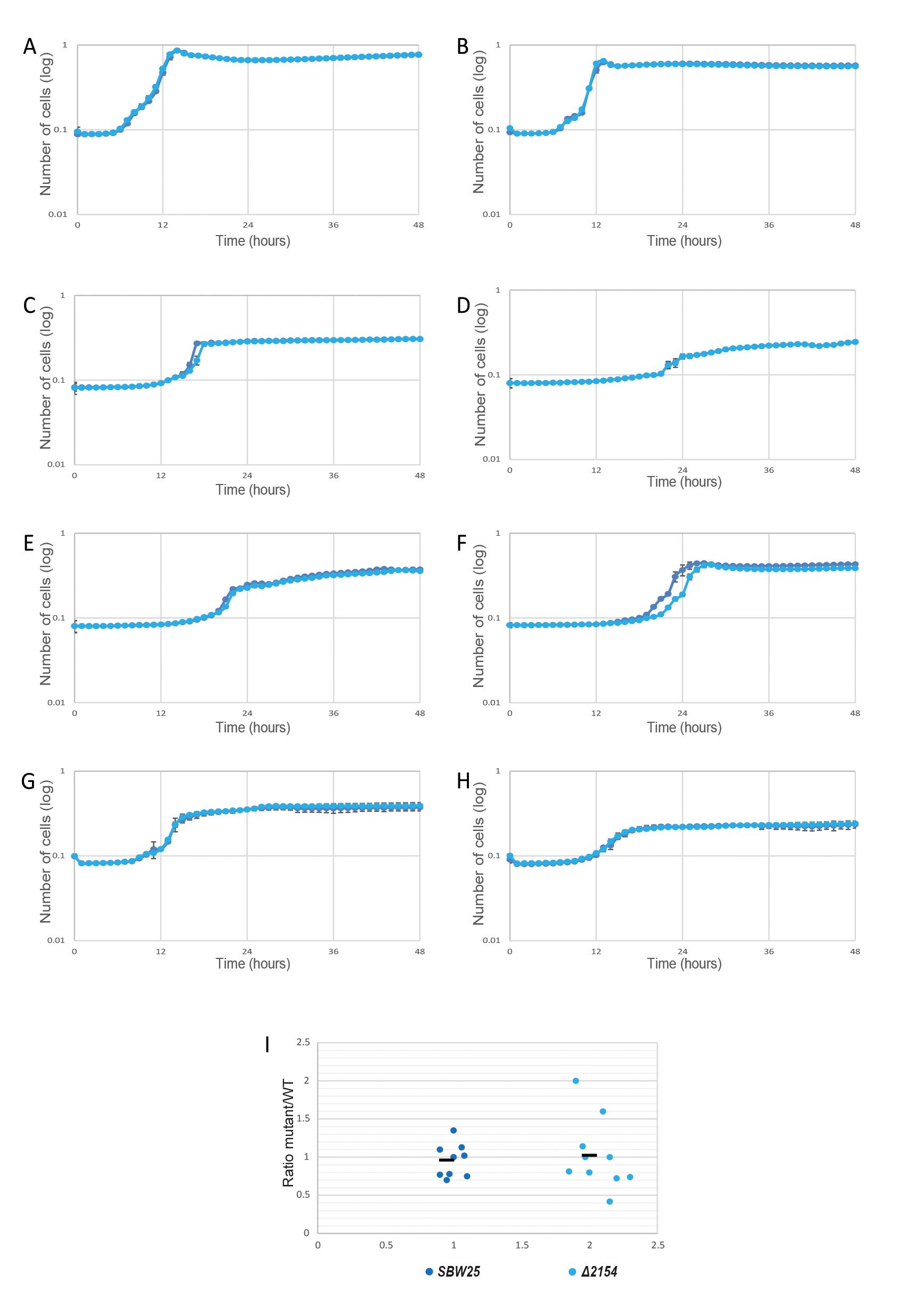

Supplement: S2 Fig — S2A : Growth curves for SBW25 and Δ2154 strains in LB, S2B: in KB media, S2C: in M9 0.4% glucose, S2D: in M9 0.4% pyruvate, S2E: in M9 0.4% glycerol, S2F: in M9 0.4% acetate, S2G: in M9 0.4% malate, and S2H: in M9 0.4% succinate. The only significant difference in growth rate was seen between WT and ΔrccR in acetate (S3F). Experiments were repeated at least three times independently. S2I: Rhizosphere colonisation competition assays. The graph shows the ratio of SBW25 WT or Δ2154 to WT-lacZ colony forming units (CFU) recovered from the rhizospheres of wheat plants seven days post-inoculation. Each dot represents CFU recovered from an individual plant. No significant differences in colonisation efficiency were seen between SBW25 and Δ2154. Experiments were repeated at least twice independently. (TIF) [file pgen.1006839.s002.tif]

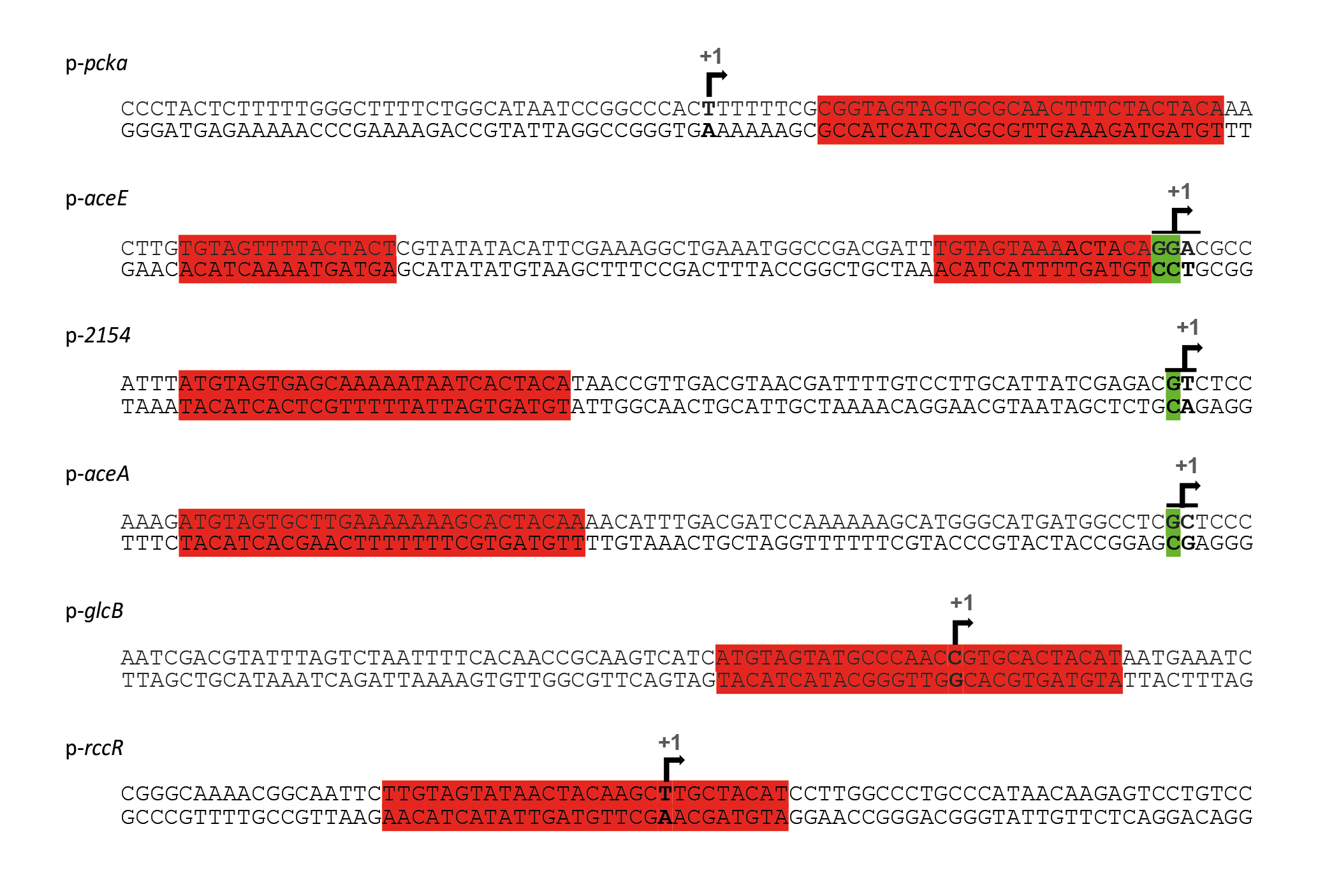

Supplement: S3 Fig — Schematic representation of pckA, aceE, PFLU2154, aceA, glcB and rccR mapping start sites. In red the RccR consensus sequences; in green the guanosines that are possible starting sites or belonging to the tagging tail. (TIF) [file pgen.1006839.s003.tif]

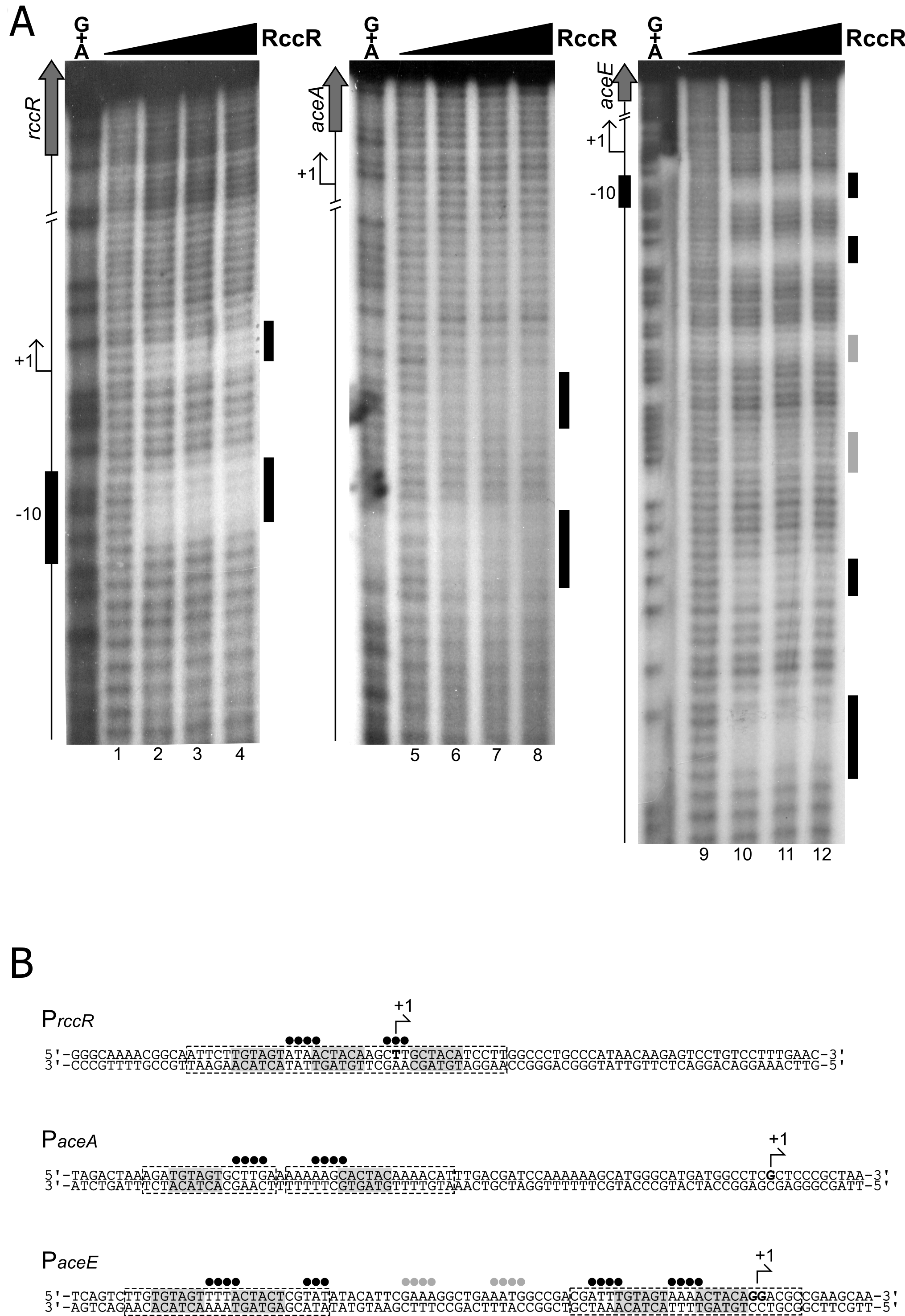

Supplement: S4 Fig — S4A: hydroxyl-radical footprintings panel of RccR on rccR, aceA and aceE promoters. RccR-His protein concentrations used in this set of experiments: 0, 40, 80, 160 nM for rccR (lanes 1 to 4) and aceE (lanes 9 to 12); 0, 80, 160, 320 nM for aceA (lanes 5 to 8). Leftmost lane of each autoradiograph: Maxam and Gilbert G+A sequence reaction ladder. Symbols are detailed in the legend of Fig 8 Panel A (in the rightmost panel, grey boxes on the right depict protected tracts in the DNA region that separates the two principal binding sites). S4B: nucleotides protected in OH-FP were mapped on the respective promoter sequences. Black/grey dots indicate nucleotides protected, while protected regions in DNaseI footprintings (Fig 8A/8B) are included in an open dashed box and conserved pseudopalindromic sequences are highlighted in light grey. Bent arrows indicate the transcriptional start sites identified in this study (S3 Fig) and the first transcribed nucleotides are in bold. (TIF) [file pgen.1006839.s004.tif]

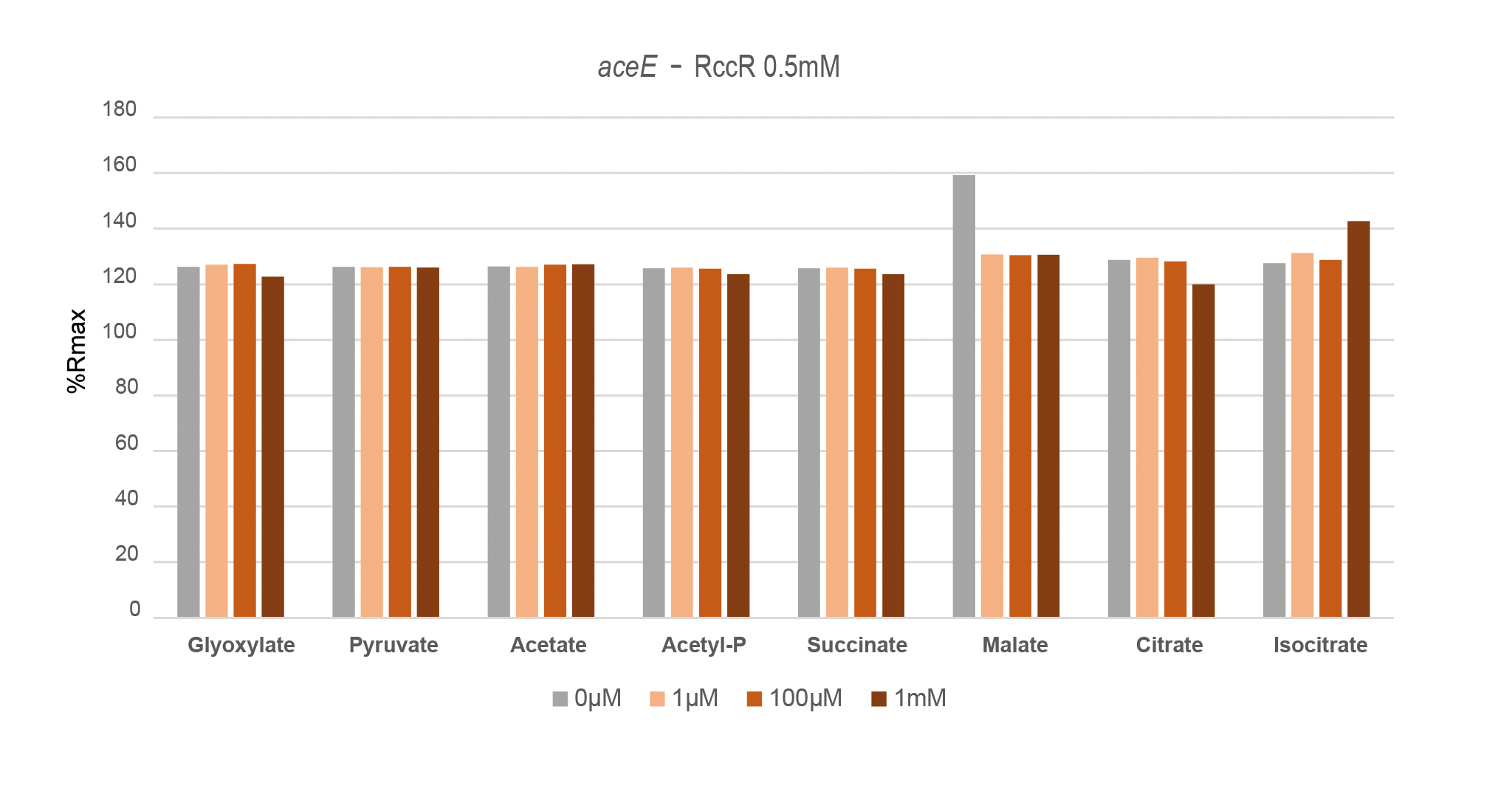

Supplement: S5 Fig — (TIF) [file pgen.1006839.s005.tif]

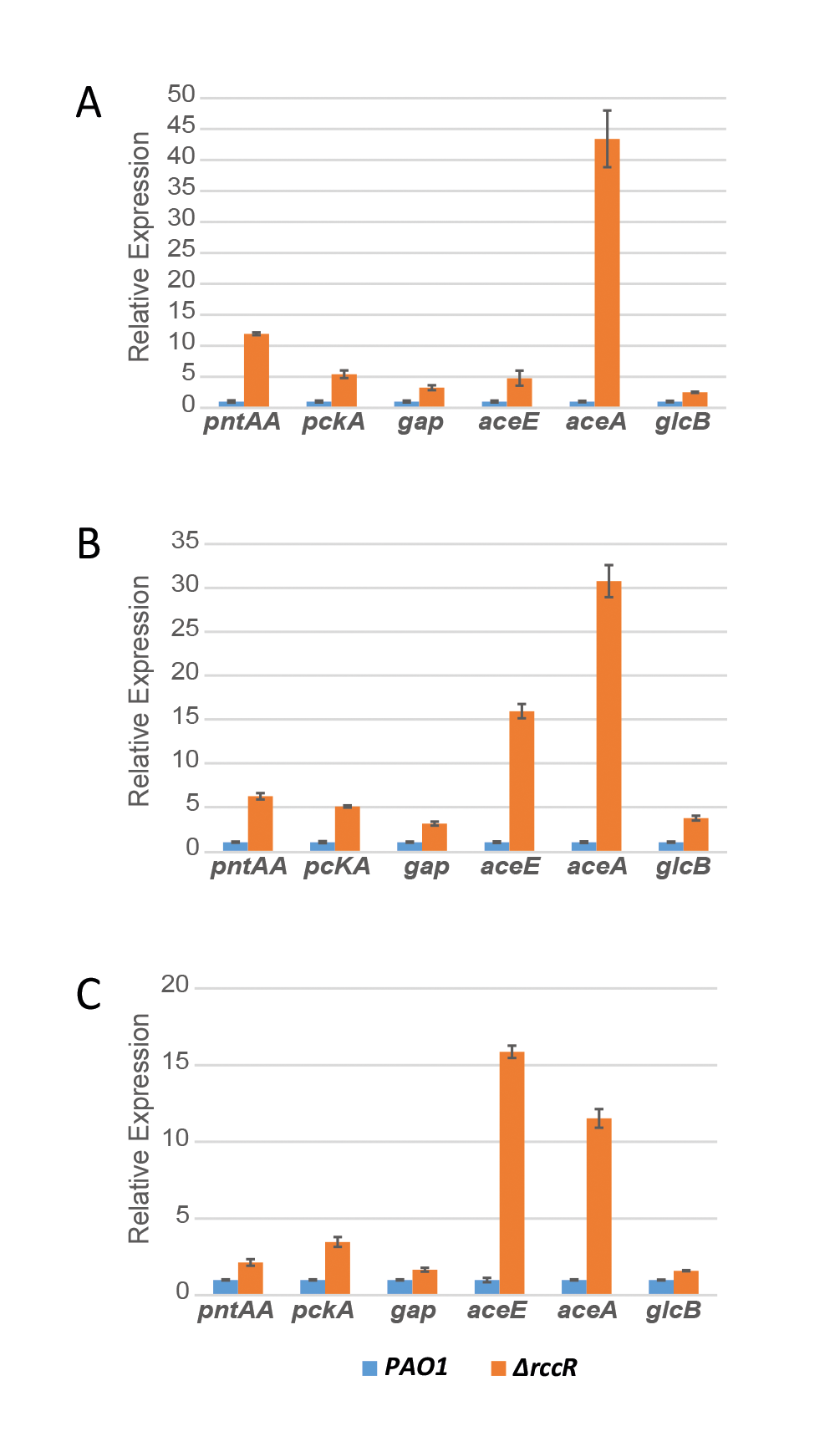

Supplement: S6 Fig — The RccR regulon in Pseudomonas aeruginosa S6A-C: PA01 RccR gene target expression determined by qRT-PCR. Data are shown for PA01 ΔrccR relative to WT in S6A: glycerol media, S6B: pyruvate media, and S7C: in acetate media. (TIF) [file pgen.1006839.s006.tif]
