# Supplementary material for: One ligand, two regulators and three binding sites: How KDPG controls primary carbon metabolism in Pseudomonas
Source: PLoS Genet. 2017 Jun 28;13(6):e1006839. doi: 10.1371/journal.pgen.1006839 (PMC5489143; doi:10.1371/journal.pgen.1006839)
Supplement: S3 Table — (DOCX) [file pgen.1006839.s009.docx]

Supp. Table 3

| **RccR gene targets** | **Strand** | **RccR consensus sequences** |
| --- | --- | --- |
| PFLU0113 *pntAA* | - | TTGTAGTTAATTTTTCGTCACCCGTCAT |
| PA0195 *pntAA* | +  + | TTGTAGTCAAAGTTTTACTTTGCTACTT TTGTAGTTTCTCTGTCGTATGCCTTCAG |
| PFLU0267 *pckA* | + | CGGTAGTAGTGCGCAACTTTCTACTACAA |
| PA5192 *pckA* | - | GTGTAGTGAGTTTTTTGAAGCGCTACGAA |
| PFLU0267 *pckA** | + | ATGTAGGGTCCGCCGAAGACCACTACCT |
| PA5192 *pckA** | - | ATGTAGGTTCCGCCGAGGCTTACTACCT |
| PFLU0460 *aceE* | - | TGTAGTTTTACTACT(37N)TGTAGTAAAACTACA |
| PA5015 *aceE* | + | TGTAGTTTTACTACT(37N)TGTAGTAAAACTACA |
| PFLU1566 *gap* | + | ATGTAGCCCCTTTTTTCAGCCCCTACAT |
| PA3001 *gap* | - | ATGTAGCCCCTTTTTTCATCCCCTACAT |
| PFLU2154 | - | ATGTAGTGAGCAAAAATAATCACTACAT |
| PFLU3817 *aceA* | + | ATGTAGTGCTTGAAAAAAAGCACTACAA |
| PA2634 *aceA* | + | GTGTAGTAGTCTTGAAAAAACACTACAA |
| PFLU5623 *glcB* | + | ATGTAGTATGCCCAACCGTGCACTACAT |
| PA0482 *glcB* | - | ATGTAGTATGCCGCGGCTCGGACTACAA |
| PFLU6073 *rccR* | - | TTGTAGTATAACTACAAGCTTGCTACAT |
| PA5438 *rccR* | - | TTGTAGTATAACTACAAGGTTACTACAG |
