# Supplementary material for: One ligand, two regulators and three binding sites: How KDPG controls primary carbon metabolism in Pseudomonas
Source: PLoS Genet. 2017 Jun 28;13(6):e1006839. doi: 10.1371/journal.pgen.1006839 (PMC5489143; doi:10.1371/journal.pgen.1006839)
Supplement: S4 Table — (DOCX) [file pgen.1006839.s010.docx]

**Supp. table 4 – Strains and Plasmids**

| Strains | Description | Reference |
| --- | --- | --- |
| Pseudomonas |  |  |
| SBW25 | Environmental *P. fluorescens* isolate | (48) |
| SBW25 ∆rccR | SBW25 with *rccR* (*PFLU_6073*) deleted | This study |
| SBW25 ∆hexR | SBW25 with *hexR* (*PFLU_4840*) deleted | This study |
| SBW25 ∆rccR∆hexR | SBW25 with *rccR, hexR* (*PFLU_6073, PFLU_4840*) deleted | This study |
| SBW25 pUCTn7Gm-lacZ | SBW25 carrying the pUCTn7Gm-*lacZ* plasmid used as control in the β-galactosidase assay | This study |
| SBW25∆rccR pGm-rccR-lacZ | SBW25∆*rccR* carrying pGm-*rccR-lacZ* plasmid used for the *rccR* gene expression study (β-galactosidase assay) | This study |
| SBW25∆hexR pGm-hexR-lacZ | SBW25∆*hexR* carrying pGm-*hexR-lacZ* plasmid used for the rccR gene expression study (β-galactosidase assay) | This study |
| PA01 | Wild-type *P. aeruginosa* | (49) |
| PA01 ∆rccR | PA01 with *rccR* (*PA5438*) deleted | This study |
| E. coli |  |  |
| DH5α  BL21(D3) | *endA*1, *hsdR*17(r_K_-m_K_+), *supE*44, *recA*1, *gyrA* (Nal^r^), *relA*1, Δ(*lacIZYA-argF*)U169, *deoR*, Φ80*dlacΔ(lacZ)M15*  SmR , K12 recF143 *lacIq lacZ*Δ.M15, *xylA* | (50)  Novagen |
| Plasmids |  |  |
| pME3087 | Tet^R^, suicide vector; ColE1-replicon, IncP-1, Mob | (51) |
| pTS-1 | pME3087 derivative containing a *sacB* counter-selection marker | T. Scott - gift |
| pTS-1- rccR/hexR vectors | pTS-1 with *rccR/hexR* SBW25 alleles as *XhoI-Bam*HI fragments amplified by PCR with oligonucleotides 1-2/3-4 and 5-6/7-8 respectively | This study |
| pTS-1- PAOrccR | pTS-1 with *rccR* PAO1 allele as *XhoI-Bam*HI fragments amplified by PCR with oligonucleotides 11-12/13-14 | This study |
| pTS-1-2154 | pTS-1 with *PFLU2154* SBW25 allele as *XhoI-Bam*HI fragments amplified by PCR with oligonucleotides 17-18/19-20 | This study |
| pUCTn7Gm-lacZ | Gm^R^, transcriptional fusion vector | (52) |
| pGm-rccR-lacZ | pUCTn7Gm-*lacZ* derivative, containing *rccR* promoter fused to *lacZ,* as *NcoI-BamHI* fragment amplified by PCR with oligonucleotides 23-24 | This study |
| pGm-hexR-lacZ | pUCTn7Gm-*lacZ* derivative, containing *hexR* promoter fused to *lacZ*, as *NcoI-BamHI* fragment amplified by PCR with oligonucleotides 25-26 | This study |
| pET42b | Kc^R^, purification vector, His6-tag | Novagen |
| pET42b-rccR | pET42b derivative with *rccR* as XhoI-NdeI fragment amplified by PCR with oligonucleotides 77-78 | This study |
| pGEM | Amp^R^; cloning vector | Promega |
| pGEM-rccR | pGEM-T-Easy derivative, containing *rccR* upstream region amplified by PCR with oligonucleotides 85-86 | This study |
| pGEM-aceA | pGEM-T-Easy derivative, containing *aceA* upstream region amplified by PCR with oligonucleotides 81-82 | This study |
| pGEM-aceE | pGEM-T-Easy derivative, containing *aceE* upstream region amplified by PCR with oligonucleotides 83-84 | This study |
| pGEM-OHrccR | pGEM-T-Easy derivative, containing *rccR* upstream region amplified by PCR with oligonucleotides 91-92 | This study |
| pGEM-OHaceA | pGEM-T-Easy derivative, containing *aceA* upstream region amplified by PCR with oligonucleotides 87-88 | This study |
| pGEM-OHaceE | pGEM-T-Easy derivative, containing *aceE* upstream region amplified by PCR with oligonucleotides 89-90 | This study |

The pTS-1 plasmid was the kind gift of Thomas Scott.
