# Supplementary material for: One ligand, two regulators and three binding sites: How KDPG controls primary carbon metabolism in Pseudomonas
Source: PLoS Genet. 2017 Jun 28;13(6):e1006839. doi: 10.1371/journal.pgen.1006839 (PMC5489143; doi:10.1371/journal.pgen.1006839)
Supplement: S5 Table — (DOCX) [file pgen.1006839.s011.docx]

**Supp. table 5 – Primers**

| Number/name | Sequence (5’→3’) |
| --- | --- |
| 1/PFLU6073DelUpF | CGGGATCCTCAGCCTGGTGCGCCAAG |
| 2/PFLU6073DelUpR | CGTCTAGATTGCAACAGGTTCAAAG |
| 3/PFLU6073DelDownF | CGTCTAGAGCCCTCGACGACTAGC |
| 4/PFLU6073DelDownR | CGGAATTCCGGTCAACATGGAATTG |
| 5/hexRDelUpF | CGGGATCCCGGCTTTCATGAAGTCG |
| 6/hexRDelUpR | CGTCTAGAGCACGCGGTCCATTC |
| 7/hexRDelDownF | CGTTAGACGAGTTCAACTGAGCC |
| 8/hexRDelDownR | CGGAATTCGGCGTATTGGTCACG |
| 9/hexRDelScrF | GCATCGTTGACCTTGCGC |
| 10/hexRDelScrR | GCAATTGCAGCTGTGCG |
| 11/PA5438DelUpF | CGGGATCCCGAGGCCGGCGATGGAGAGGT |
| 12/PA5438DelUpR | GCTCTAGAGTTCACAGGCTGGGCTCGATT |
| 13/PA5438DelDowF | GCTCTAGATCCTGAGGCGCCGCCGGGAAG |
| 14/PA5438DelDowR | CCGCTCGAGCTGGCCCTGCTGCCGCGCCAT |
| 15/PA5438sceF | AAGAACTCCTCGCAAGCCTTG |
| 16/PA5438sceR | GCGCGCATCGTGCTGGAAAAA |
| 17/2154DelUpF | CGGGATCCCCACATTGAACGGTGTTGATG |
| 18/2154DelUpR | GCTCTAGAAAGCCCCTTGACCAGTTGGCG |
| 19/2154DelDoF | GCTCTAGAACTGCTGAACACTAAGAAACT |
| 20/2154DelDoR | CCGCTCGAGAATCGATTGGAAGACCTTGC |
| 21/2154DelScrF | CGACACGCTGGAACATC |
| 22/2154DelScrR | TGTGGTGCCCGGTGATT |
| 23/6073PromF | GCGGTACCTTCCTGAGTGATGCC |
| 24/6073PromR | CTGGATCCCTAGTCGTCGAGGGCTTTGACGG |
| 25/hexRPromF | CATGCCATGGGATGTTTGTTCTTTTATCAAGT |
| 26/hexRPromR | CGGGATCCTCAGTCAGTCTTTCCTTCGGGC |
| 27/pTS-1screenF | CGGCAGGTATATGTGATGG |
| 28/pTS-1screenR | GTGAGAAATCACCATGAGTG |
| 29/qhexR_F | ATCTTTGGCAGCGCCAT |
| 30/qhexR_R | AACTTGTGCAGCGCAT |
| 31/q6073_F | GCGAGCTTCGGGCAATTTGC |
| 32/q6073_R | CCATAGAACTCAACGCGCTG |
| 33/qzwfSBW_F | AATACGTCGGCAAGGAACTG |
| 34/qzwfSBW_R | CACGTAGTCATCGGCTTTCA |
| 35/qgap1SBW_F | CTTCAACCATAACCCGCTGT |
| 36/qgap1SBW_R | AGCCCCACTCGTTGTCATAC |
| 37/qeddSBW_F | GGCACCGAAGACAAAAACAG |
| 38/qeddSBW_R | GAAATGTTCGTACGGCTGGT |
| 39/qgltSBW_F | CCTGGTCATCCTTGATGTGA |
| 40/qgltSBW_R | CATGATGATCGGCACCTG |
| 41/qPFLU0113_F | TCGTGGACAATGCCTATGAA |
| 42/qPFLU0113_R | GGTTTCATTGCTGAACGGAT |
| 43/qPFLU0267_F | CGACGTACTGCCGATGC |
| 44/qPFLU0267_R | AGCCGTGTTCGTCGTCA |
| 45/qPFLU0460_F | CACTGCGCACAACACCA |
| 46/qPFLU0460_R | CGCTCAGGTAACGGGCT |
| 47/qPFLU0461_F | GCGCATTGCCTTTATGCT |
| 48/qPFLU0461_R | CCACGACCAGTTCGCTCT |
| 49/qPFLU1565_F | TGATCCCCGAAGACTACCTG |
| 50/qPFLU1565_R | TTCTTGATCCCCAATTGCTC |
| 51/qPFLU1566_F | ATCGCGATTCTCAAAGCG |
| 52/qPFLU1566_R | CCAGCTTGCCCAGGTCTA |
| 53/qPFLU2154_F | GCAATCACCGCGAACTG |
| 54/qPFLU2154_R | CGATTGGCCCTTGAGGT |
| 55/qPFLU3817_F | GACCCGTACGACCAGCC |
| 56/qPFLU3817_R | TCGTCCAGGTCCGGTTT |
| 57/qPFLU5622_F | TCGTGTGTTAGTGGGCGA |
| 58/qPFLU5622_R | CATCCGTGCTCAGGCTCT |
| 59/qPFLU5623_F | TGGTGCCGGTGATGAAC |
| 60/qPFLU5623_R | GATCACCTTGTCGCCCC |
| 61/qrpoDSB_F | CAACGAAGTAGACGAAAGCTC |
| 62/qPA5192F | TTCTCCGTGCAGAACTTCCT |
| 63/qPA5192R | CCGAAGAACAGGGTCACATC |
| 64/qPA5051F | CAGCTCGGCATCTACTCCTC |
| 65/qPA5051R | GAGGATCTGGCCCTTCTTGT |
| 66/qPA3001F | CGAAGAAAACAACACGCTGA |
| 67/qPA3001R | TTGATGCCGTACTGGGTGTA |
| 68/qPA2634F | TCCTCCGTCTACACCCAGTC |
| 69/qPA2634R | TTGTTGGTGGTCTTCAGGTG |
| 70/qPA0482F | GACGAGATCCACACCTCCAT |
| 71/qPA0482R | GACGTTGTTGTTCTCGTAGGC |
| 72/qPAO0195F | AACCGTCAAGAAGCTGATCG |
| 73/qPAO0195R | CGGCTTCATAGGCACTGTC |
| 74/qPAO1rpoD_F | CGCCAAGAAGTACACCAACC |
| 75/qPAO1rpoD_R | GCGACGGTATTCGAACTTGT |
| 76/qrpoDSB_R | GACGGTTGATGTCCTTGATCTC |
| 77/rccRclon_F | GACATATGAACCTGTTGCAACATATCG |
| 78/rccRclon_R | TCCTCGAGGTCGTCGAGGGCTTTGACG |
| 79/rccRcom_F | CGGAATTCTTGAACCTGTTGCAACATATCG |
| 80/rccRcom_R | GGGGTACCGCTTTGTTTAGCAGCCTAGGT |
| 81/aceA_F | CCTTGAACTGAAGCACCAAA |
| 82/aceA_R | TACAGACACGAATCGGCTCA |
| 83/aceE_F | GATTATGCTCACCAGCCAGA |
| 84/aceE_R | AATCGGTTGCCTGAAGTTTG |
| 85/rccR_F | ACAACTTGGCTGCCCTTAAA |
| 86/rccR_R | GTTCCGATTTGCGTAACAGG |
| 87/aceAOH_F | ATATGGATCCATGAGTCGTCTTAGACTAAAGATG |
| 88/aceAOH_R | ATATCTCGAGTTTGGATCGTCAAATGTTTTGTAG |
| 89/aceEOH_F | ATATGGATCCAGCCATACGCTCAGTCTTGTG |
| 90/aceEOH_R | ATATCTCGAGAAGGTTGCTTCGGCGTCCTG |
| 91/rccROH_F | ATATGGATCCACGGGCAAAACGGCAATTCTTG |
| 92/rccROH_R | ATATCTCGAGTGTTATGGGCAGGGCCAAGGATG |
| 93/pntAASPR_F | CTTGTAGTTAATTTTTCGTCACCCGTCATA |
| 94/pntAASPR_R | TATGACGGGTGACGAAAAATTAACTACAAcctaccctacgtcctcctgc |
| 95/pckASPR_F | CGGTAGTAGTGCGAAACTTTCTACTACAAA |
| 96/pckASPR_R | TTTGTAGTAGAAAGTTGCGCACTACTACCGcctaccctacgtcctcctgc |
| 97/aceESPR_F | CTTGTGTAGTTTTACTACTCGTATATACAT |
| 98/aceESPR_R | ATGTATATACGAGTAGTAAAACTACACAAcctaccctacgtcctcctgc |
| 99/gapSPR_F | AATGTAGCCCCTTTTTTCAGCCCCTACATG |
| 100/gapSPR_R | CATGTAGGGGCTGAAAAAAGGGGCTACATTcctaccctacgtcctcctgc |
| 101/aceASP_F | TATGTAGTGAGCAAAAATAATCACTACATA |
| 102/aceASPR_R | TATGTAGTGATTATTTTTGCTCACTACATAcctaccctacgtcctcctgc |
| 103/2154SPR_F | GATGTAGTGCTTGAAAAAAAGCACTACAAA |
| 104/2154SPR_R | TTTGTAGTGCTTTTTTTCAAGCACTACATcctaccctacgtcctcctgc |
| 105/glcBSPR_F | CATGTAGTATGCCCAACCGTGCACTACATA |
| 106/glcBSPR_R | TATGTAGTGCACGGTTGGGCATACTACATcctaccctacgtcctcctgc |
| 107/rccRSPR_F | CTTGTAGTATAACTACAAGCTTGCTACATC |
| 108/rccRSPR_R | GATGTAGCAAGCTTGTAGTTATACTACAAcctaccctacgtcctcctgc |
| 109/ctrSPR_F | TCTATCAGAGTATACACATAGATCTCTGT |
| 110/ctrSPR_R | ACAGAGATCTATGTGTATACTCTGATAGAcctaccctacgtcctcctgc |
| 111/267GSP-1 | AGGAAGTTCTGCACCGAGAA |
| 112/267GSP-2 | TTGAGCACTTGCCACTCATC |
| 113/267GSP-3 | CGGGTTGTATTTCGCCGGGTT |
| 114/460GSP-1 | GGAAGCACCAGACTTTCTGC |
| 115/460GSP-2 | CATGTGTTCTTCGGTGATGC |
| 116/460GSP-3 | CTGGAAGTAGATCAGGTCGCCG |
| 117/2154GSP-1 | TTACCTTAACGTGCGATTGG |
| 118/2154GSP-2 | AACTCGGCCTCTTCGGTATT |
| 119/2154GSP-3 | TGCACTTCGCCCGATTTGCC |
| 120/3817GSP-1 | GCCTCGATCATGCTCTTCAT |
| 121/3817GSP-2 | GTCGGGGTACATGGATTCAG |
| 122/3817GSP-3 | TTACCTGTTGGACTGCCTGGCC |
| 123/5623GSP-1 | TTGCGGTAGATCACCACTTTG |
| 124/5623GSP-2 | CCATACAGCGCGTCATACAG |
| 125/5623GSP-3 | GCCATGCGGGCGATTTCGTCAT |
| 126/6073GSP-1 | GGTGATCAGCAGGTCTTTGG |
| 127/6073GSP-2 | GCACCGAAGCCATAGAACTC |
| 128/6073GSP-3 | CAGGGTGGTGTCGAAGATTT |
